# The glucocorticoid receptor acts locally to protect dystrophic muscle and heart during disease

**DOI:** 10.1242/dmm.050397

**Published:** 2024-05-21

**Authors:** Trinitee Oliver, Nhu Y. Nguyen, Christopher B. Tully, Nikki M. McCormack, Christina M. Sun, Alyson A. Fiorillo, Christopher R. Heier

**Affiliations:** ^1^ Center for Genetic Medicine Research, Children's National Hospital, Washington, DC 20010, USA; ^2^ Department of Biology, Howard University, Washington, DC 20059, USA; ^3^ Graduate School of Biomedical Sciences, Cedars-Sinai Medical Center, West Hollywood, CA 90048, USA; ^4^ Department of Molecular Biomedical Sciences, North Carolina State University, Raleigh, NC 27607, USA; ^5^ Department of Genomics and Precision Medicine, The George Washington University, Washington, DC 20037, USA; ^6^ Center for Inherited Muscle Research, Department of Neurology, Virginia Commonwealth University, Richmond, VA 23298, USA

**Keywords:** Steroids, Muscular dystrophy, Mouse model, Inflammation, Muscle, Heart

## Abstract

Absence of dystrophin results in muscular weakness, chronic inflammation and cardiomyopathy in Duchenne muscular dystrophy (DMD). Pharmacological corticosteroids are the DMD standard of care; however, they have harsh side effects and unclear molecular benefits. It is uncertain whether signaling by physiological corticosteroids and their receptors plays a modifying role in the natural etiology of DMD. Here, we knocked out the glucocorticoid receptor (GR, encoded by *Nr3c1*) specifically in myofibers and cardiomyocytes within wild-type and *mdx52* mice to dissect its role in muscular dystrophy. Double-knockout mice showed significantly worse phenotypes than *mdx52* littermate controls in measures of grip strength, hang time, inflammatory pathology and gene expression. In the heart, GR deletion acted additively with dystrophin loss to exacerbate cardiomyopathy, resulting in enlarged hearts, pathological gene expression and systolic dysfunction, consistent with imbalanced mineralocorticoid signaling. The results show that physiological GR functions provide a protective role during muscular dystrophy, directly contrasting its degenerative role in other disease states. These data provide new insights into corticosteroids in disease pathophysiology and establish a new model to investigate cell-autonomous roles of nuclear receptors and mechanisms of pharmacological corticosteroids.

## Teaser

**Summary:** Muscle-specific knockout of glucocorticoid receptor (GR) in a Duchenne muscular dystrophy (DMD) mouse model shows that physiological GR exerts a protective role in DMD, contrasting its degenerative role in other diseases.

## INTRODUCTION

Progressive muscle weakness, chronic inflammation and cardiomyopathy are characteristic features of Duchenne muscular dystrophy (DMD), an X-linked muscle disease resulting from a loss of dystrophin function (Hoffman et al., 1987; Koenig et al., 1987; Monaco et al., 1986). Dystrophin-deficient muscle begins to display immune-like gene expression changes, such as active NF-κB signaling and myofiber-localized toll-like receptors, years before symptom onset (Chen et al., 2005; Porter et al., 2002, 2003). Pharmacological glucocorticoids, including prednisone or deflazacort, are the standard of care in DMD, and we hypothesize that they act locally within myofibers to suppress pathological inflammatory signaling. However, treating children chronically with these drugs results in harsh side effects that negatively impact patient quality of life. These harsh side effects also obscure the mechanism of action by which glucocorticoids benefit patients with DMD, with some postulating that it is the side-effect pathways (e.g. stunted growth) that provide benefit. Thus, defining the role of the glucocorticoid receptor (GR; encoded by *Nr3c1* in mice) in the natural etiology of muscular dystrophy will help to clarify the mechanism of action for this drug class and to develop improved treatments for muscle diseases featuring chronic inflammation.

In individuals without dystrophy, prolonged exposure to glucocorticoids can cause muscle atrophy and weakness (Ma et al., 2003; Seene, 1994), which is in stark contrast to the strength increases seen in DMD and has led to debate about their mechanism of action. Most directly, a hormonal disorder named Cushing's syndrome presents with muscle weakness in 60-82% of patients and is caused by excess glucocorticoids, either pharmacological prednisone or physiological cortisol (reviewed in Braun et al., 2019). Coincident increases in physiological glucocorticoids are also associated with muscle weakness in diabetes mellitus (Hu et al., 2009), sepsis (Voisin et al., 1996), starvation (Wing et al., 1995) and metabolic acidosis (May et al., 1986). The role of glucocorticoids in myopathy for these systems is driven by transactivation properties of the GR (where the GR functions as a transcription factor), which upregulates muscle atrophy and protein catabolism pathways such as the E3 ubiquitin ligases muscle atrophy F-box (MAFbx, encoded by *Fbxo32*) and muscle RING finger 1 (MuRF1, encoded by *Trim63*) (Bodine et al., 2001). We found that off-target transactivation pathways such as these could be dissociated from anti-inflammatory properties (e.g. inhibition of NF-κB) of GR ligands by adopting a Δ9,11 structure, which provides a mechanistic basis to develop improved drugs with reduced side effects (Fiorillo et al., 2018; Heier et al., 2013, 2019). It is unclear, however, what the physiological role of the GR is within the natural etiology of disease in the context of chronic muscle inflammation. In contrast to what might be expected due to its anti-inflammatory properties, recent findings suggest that the GR can also act as a key driver of inflammatory muscle wasting, as its deletion substantially improves myopathy in models of cancer- and endotoxin-induced cachexia (Braun et al., 2013). This indicates that, in some instances, the GR participates in driving muscle weakness in diseases featuring chronic inflammation, and that inducing degradation of the GR could act as a co-therapy for anti-inflammatory drugs. Further investigations in DMD and comparable inflammatory muscle diseases are needed to determine whether the GR protects against or whether it aggravates disease course.

Additional effects of pharmacological glucocorticoids in DMD include stunted growth and immunosuppression. Some groups have proposed that impaired growth of boys with DMD (between 7 and 18 years of age) may actually be a pathway of efficacy by limiting muscle workload and delaying muscle maturation (Grounds and Shavlakadze, 2011). However, the novel GR ligand vamorolone increases strength in *mdx* mice and boys with DMD without causing the same stunted growth as that seen for prednisone (Heier et al., 2013; Smith et al., 2020; Mah et al., 2022; Dang et al., 2024). Alternatively, substantial efforts have been pursued to develop non-steroidal immunosuppressive or anti-inflammatory treatment options. However, immunosuppressive drugs such as azathioprine may reduce inflammation but fail to increase the strength of patients with DMD (Griggs et al., 1993; Kissel et al., 1993). Anti-inflammatory drugs that have alternative targets, such as the NF-κB inhibitor edasalonexent or the anti-TNF biologic Remicade, have shown promise in animal models but failed to show efficacy in DMD clinical trials (Grounds and Torrisi, 2004; Hammers et al., 2016; Finkel et al., 2021). Elucidating how and where the GR impacts dystrophic muscle during disease etiology will help clarify whether growth and immune cells, as opposed to muscle itself, are relevant targets for the treatment of muscular dystrophy.

Early attempts to study GR pathways in disease models were made difficult by the perinatal lethality of homozygous GR deletion (Cole et al., 1995); however, tissue-specific knockout mice can now be used to study muscle (Braun et al., 2013; Watson et al., 2012), immune (Tuckermann et al., 2007; Brewer et al., 2003), heart (Oakley et al., 2019, 2013), osteoblast (Rauch et al., 2010) and neuronal cells (Tronche et al., 1999; Boyle et al., 2005). Here, we created a novel double-knockout (dKO) *mdx* mouse model featuring muscle-specific knockout of the GR in the *mdx52* mouse model of DMD, which features a deletion of murine *Dmd* exon 52 that causes absence of the dystrophin protein. In comparison to *mdx52* littermate controls, the resulting dKO mice showed consistently worse phenotypes in measures of muscle weakness, inflammation and cardiomyopathy. These studies establish these dKO mice as a novel system with which to study glucocorticoid signaling in muscular dystrophy. Data show that the GR acts locally to provide a protective function for both heart and skeletal muscle during natural disease etiology in DMD.

## RESULTS

### Generation of tissue-specific dKO mice

**Fig. 1.**
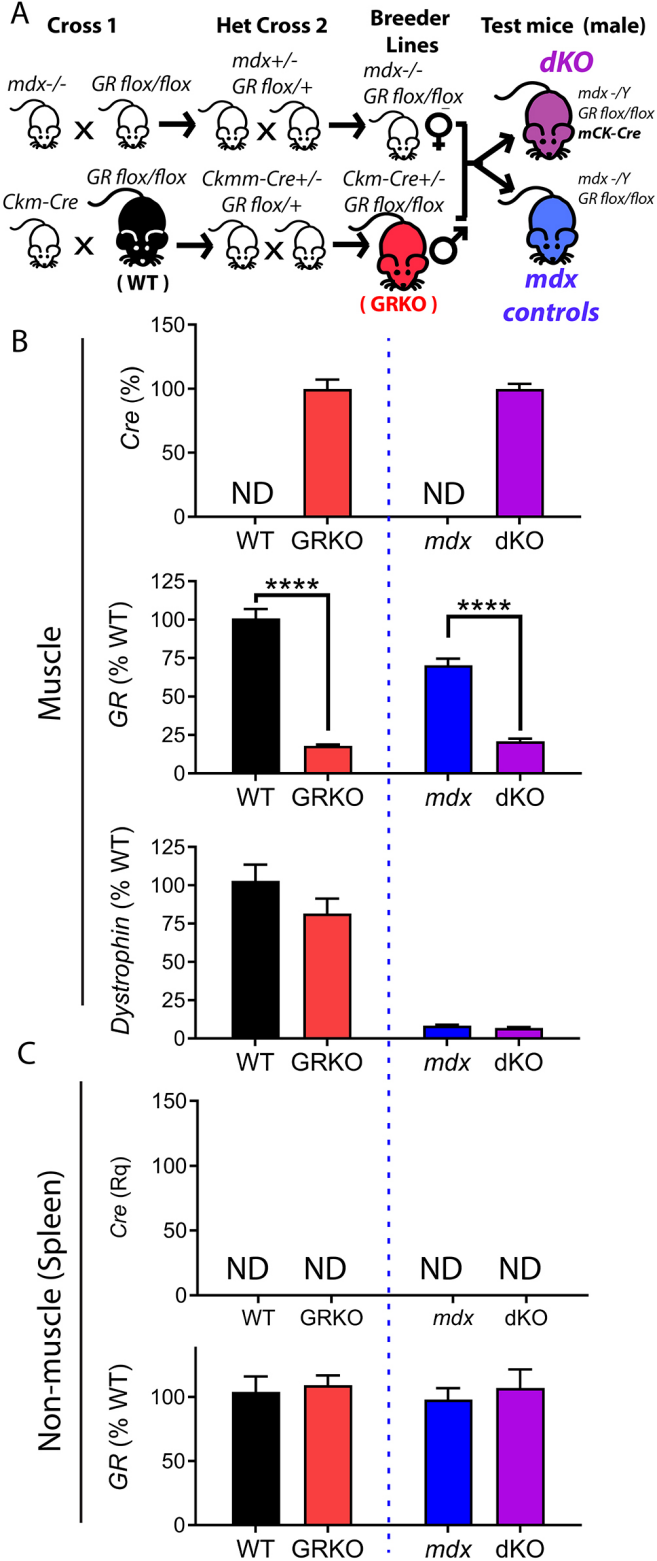
**Generation of tissue-specific double-knockout mice.** (A) Breeding scheme used to establish double-knockout (dKO) mice. Genotypes relevant to experiments are denoted and color-coded in the schematic; genotypes used as wild-type (WT) and glucocorticoid receptor (GR) knockout (GRKO) are depicted within the dKO scheme here, but they were maintained as a separate line of mice homozygous for *GR^lox^* and either hemizygous or absent for Cre. (B) qRT-PCR of muscle (gastrocnemius) from 3-month-old mice confirmed expression of Cre, knockdown of GR and absence of dystrophin expression. (C) qRT-PCR of the spleen confirmed that non-muscle tissues lacked Cre expression and knockdown of GR. *n*=6 mice per group. Data show mean±s.e.m. ND, not detected; Rq, relative quantification. ****P≤0.0001; unpaired two-tailed *t*-test of Cre-positive versus littermate control genotypes.

To create dystrophic mice that feature tissue-specific deletion of the GR, we used a Cre/LoxP system (Fig. 1A). For this, three lines of transgenic mice were crossed with all mice maintained on a C57BL/6 background. The three transgenic lines consisted of mice with a LoxP-flanked GR allele (*GR^flox/flox^*), mice expressing Cre recombinase from a *Ckmm* promoter (*Ckmm-Cre*) that is expressed specifically in myofibers and cardiomyocytes, and the dystrophin-null *mdx52* mouse (*Dmd^−/−^*). For all experiments, Cre was maintained in a hemizygous state to avoid toxic overexpression of Cre, and so that a 50% Cre-positive rate in litters would enable GR-positive littermate controls to be compared directly to GR-knockout (GRKO) genotypes [wild type (WT) versus GRKO, and *mdx52* versus dKO].

Expression of Cre recombinase in muscle and heart tissues was validated by quantitative real-time PCR (qRT-PCR) (Fig. 1B; Fig. S1). Gene expression analysis also confirmed successful deletion of GR in Cre-positive muscle and heart tissues, as well as the absence of dystrophin in *mdx52* and dKO genotypes. To confirm Cre expression was specific to muscle, we also assayed spleen and kidney as examples of non-muscle tissues for all genotypes via qRT-PCR (Fig. 1C; Fig. S1). qRT-PCR demonstrated an absence of Cre transcripts in both spleen and kidney, and correspondingly showed GR expression levels similar to those in WT.

**Fig. 2.**
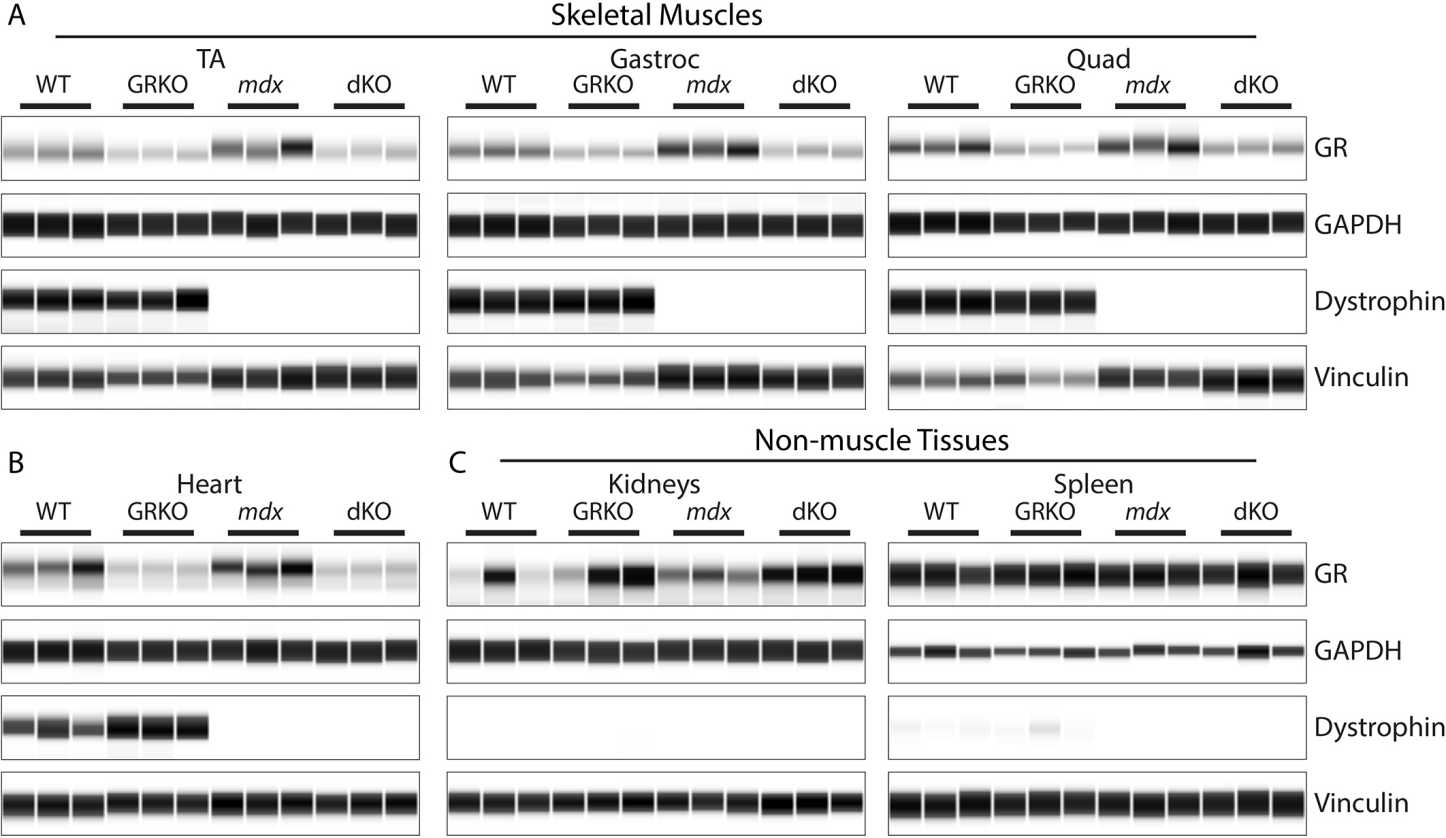
**Tissue-specific knockout of the GR protein in dKO muscle and heart.** GR and dystrophin protein levels were assayed via capillary western immunoassay in both muscle and non-muscle tissues for the indicated genotypes. Sample results from three mice per genotype are shown. GAPDH and vinculin are included as loading controls. (A) Successful knockout of the GR was observed in skeletal muscle. (B) Heart muscle showed successful GR knockout. (C) Non-muscle tissues (kidney and spleen) showed full expression of the GR protein. Gastroc, gastrocnemius; Quad, quadriceps; TA, tibialis anterior.

Next, we assayed the levels of GR and dystrophin at the protein level using a capillary-based western immunoassay. In both GRKO and dKO mice, all skeletal muscles examined showed clear reduction of the GR protein, including the tibialis anterior, gastrocnemius and quadriceps muscles (Fig. 2; Fig. S2). Additionally, GR knockout was confirmed in the heart of GRKO and dKO mice. Examining non-muscle tissues, we found no decrease in GR protein levels in either kidneys or spleen from GRKO and dKO mice. In the kidney, GR levels were observed to be more variable and showed potentially increased levels in both GRKO genotypes. The absence of dystrophin was confirmed in all *mdx52* and dKO skeletal muscles. The spleen showed low levels of dystrophin protein in WT and GRKO genotypes, and absence of dystrophin in *mdx52* and dKO genotypes. Kidneys showed an absence of full-length dystrophin; however, they showed high levels of the Dp71 isoform of dystrophin (Fig. S3). Expression of this non-muscle isoform in kidneys is consistent with previous reports (Duan et al., 2021; Haenggi et al., 2005; Loh et al., 2000). Taken together, these data demonstrate the successful creation of muscle-specific GR knockout mice on both WT and *mdx52* backgrounds.

**Fig. 3.**
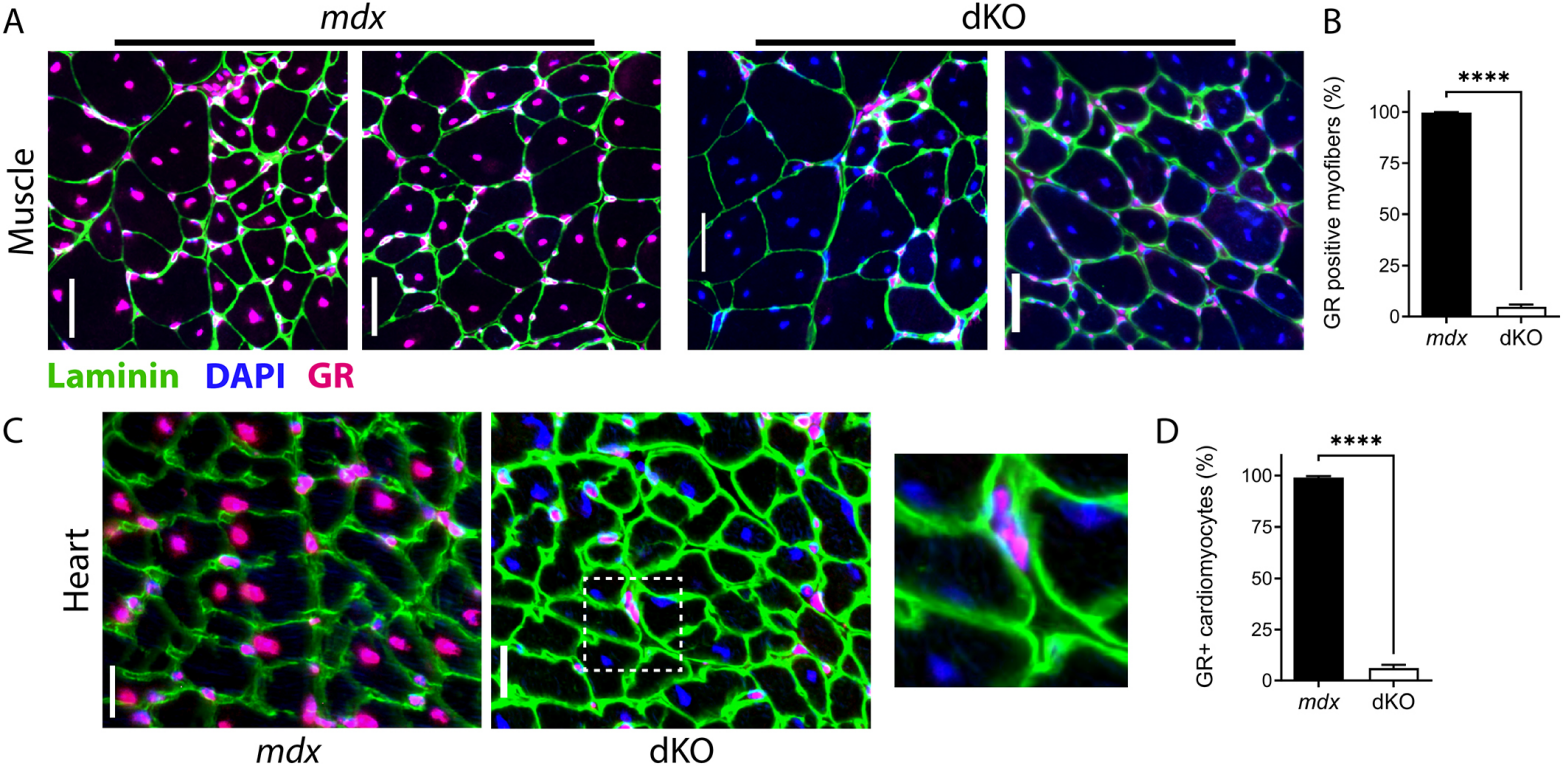
**Confirmation of GR knockout in target cell types via immunofluorescence.** GR presence or absence was assayed via immunofluorescence of skeletal muscle (gastrocnemius) and heart sections from dKO and *mdx52* littermate controls at 3 months of age. Nuclei were visualized with DAPI (blue) and myofiber boundaries were imaged with laminin (green). GR staining (magenta) was predominantly nuclear in myofibers and cardiomyocytes. (A) Successful knockout of the GR was observed in the mature myofibers of dKO mice, whereas GR protein expression was maintained in interstitial, non-myofiber cell types. (B) Quantification of the percentage of GR-positive nuclei in *mdx52* versus dKO myofibers. (C) Successful knockout of the GR in cardiomyocytes of dKO mice, with sustained protein expression in interstitial, non-cardiomyocyte cell types. An inset is provided to visualize interstitial nuclei outside of cardiomyocyte membrane boundaries. (D) Quantification of the percentage of GR-positive cardiomyocytes. *n*=3-4 mice per group. Data show mean±s.e.m. ****P<0.0001; unpaired two-tailed *t*-test. Scale bars: 50 µm (A); 20 µm (C).

To examine the efficiency of GR knockout within the target cell types of myofibers and cardiomyocytes, we performed immunofluorescence staining of skeletal muscle and heart sections. In *mdx52* littermate control mice, GR was clearly present in a majority of the nuclei in both myofibers and in interstitial cell types that are external to myofibers (Fig. 3A). GR knockout was confirmed within myofibers of dKO mice, which primarily showed only DAPI staining of nuclei, whereas interstitial cell types remained positive for immunofluorescent GR staining. Quantification showed that >99% of *mdx52* myofibers contained nuclei that stained positive for GR, whereas only approximately 5% of dKO myofibers contained nuclei that stained positive for GR (Fig. 3B). Similar findings were observed in the heart, where dKO mice showed ablation of GR in cardiomyocyte nuclei while retaining GR in interstitial cell types (Fig. 3C). Here, in *mdx52* littermate controls, 99% of cardiomyocyte nuclei were positive for GR staining, whereas this number was reduced to 6% in dKO mice (Fig. 3D). Of note, interstitial cells can contribute to residual detection of target protein presence within whole-tissue lysates of Cre systems, and this can result in increased detection for genotypes with increased interstitial cells such as *mdx*. These data illustrate that the GR is successfully ablated from the target cell types of myofibers and cardiomyocytes.

To determine whether muscle knockout of GR impacted systemic levels of cytokines or physiological glucocorticoids, we performed enzyme-linked immunosorbent assay (ELISA) analyses of mouse serum samples (Fig. S4). We found no significant difference between genotypes for IL-6 levels. We also observed no significant difference for levels of corticosterone, the physiological corticosteroid in mice, with all genotypes showing values between 50 and 150 ng/ml. To determine whether this physiological level is consistent with the ability of corticosterone to inhibit inflammatory signaling, we performed an *in vitro* assay. Here, corticosterone concentrations of 10, 100 and 1000 ng/ml showed significant inhibition of an NF-κB luciferase reporter. Taken together, these data show that circulating levels of glucocorticoids and cytokines are maintained in dKO mice, and that physiological corticosterone levels are consistent with concentrations that can affect inflammatory signaling.

### The GR acts directly within muscle to protect it against dystrophy

**Fig. 4.**
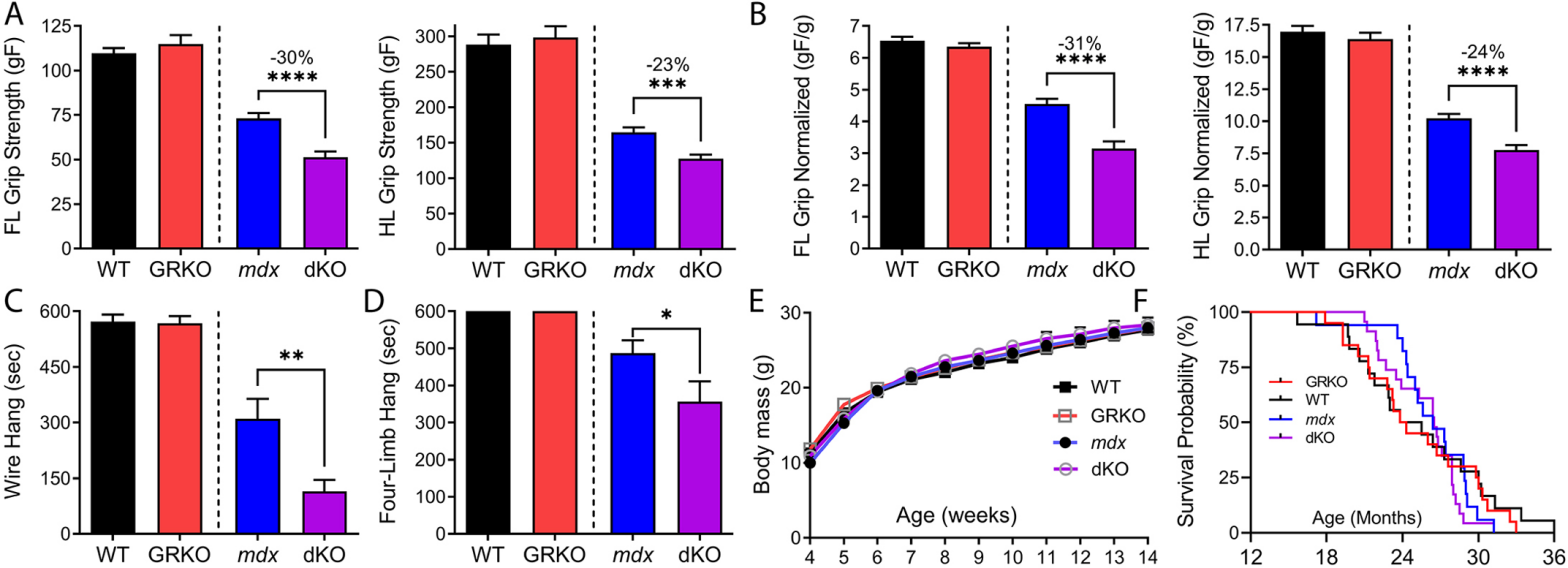
**The GR acts within myofibers to prevent further strength loss in muscular dystrophy.** Strength of dKO mice was assessed through three phenotyping assays beginning at 5 weeks of age (*n*≥12 mice per group). (A,B) Grip strength at 5 weeks of age showed a significant decrease in dKO mice versus *mdx52* littermate controls for both forelimbs (FL) and hindlimbs (HL) in absolute (A) and weight-normalized (B) values. gF, grams-force. (C,D) Both the two-limb wire hang at 7 weeks (C) and four-limb grid hang at 8 weeks (D) of age showed significant decreases in function for dKO mice versus *mdx52* littermate controls. (E) Graph of body mass over time, through phenotyping assays to 14 weeks of age. (F) Survival of the four genotypes of mice followed through the full mouse lifespan (*n*≥17 per group). Data show mean±s.e.m. *P≤0.05; **P≤0.005; ***P≤0.0005; ****P≤0.0001; unpaired two-tailed *t*-test of Cre-positive versus control littermate genotypes.

To determine whether the GR plays a protective role within myofibers or whether it instead acts to exacerbate the progression of muscle disease, we performed phenotyping on dKO mice versus control *mdx52* littermates at 5 weeks of age. dKO mice showed reduced grip strength versus that of *mdx52* mice for forelimb [51.4 versus 73.1 gram force (gF), *P*≤0.0001] and for hindlimb [127.3 versus 164.6 gF, *P*≤0.0005) (Fig. 4A). Normalized measures of grip strength were also reduced in dKO versus *mdx52* littermates, with both forelimb (31% decrease, *P*≤0.0001) and hindlimb (24% decrease, *P*≤0.0001) showing significant reductions (Fig. 4B). Reduced suspension times were also found using wire-hang (*P*≤0.01) and grid-hang (*P*≤0.05) strength-phenotyping assays (Fig. 4C,D). These data support the hypothesis that physiological GR provides a protective function that helps to maintain mouse strength during the etiology of natural disease in dystrophic *mdx* mice.

Despite the impact of GR deletion on *mdx52* skeletal muscle phenotypes, we found no clear impact of GR deletion on the body weight or the survival of mice. Examining body weights, we saw no significant differences between genotype groups through 14 weeks of age, which includes the age ranges for which phenotyping assays were performed (Fig. 4E). Although a subset of dKO mice may have shown a slightly earlier onset of mortality (∼1-2 months) than that for *mdx52* mice, these two genotypes showed similar medians and both showed a maximal survival of 31 months, with no significant difference between the curves (Fig. 4F). No impact of single GR knockout was detected on the survival of GRKO versus WT mice, which showed very similar survival curves and survived up to 36 months.

**Fig. 5.**
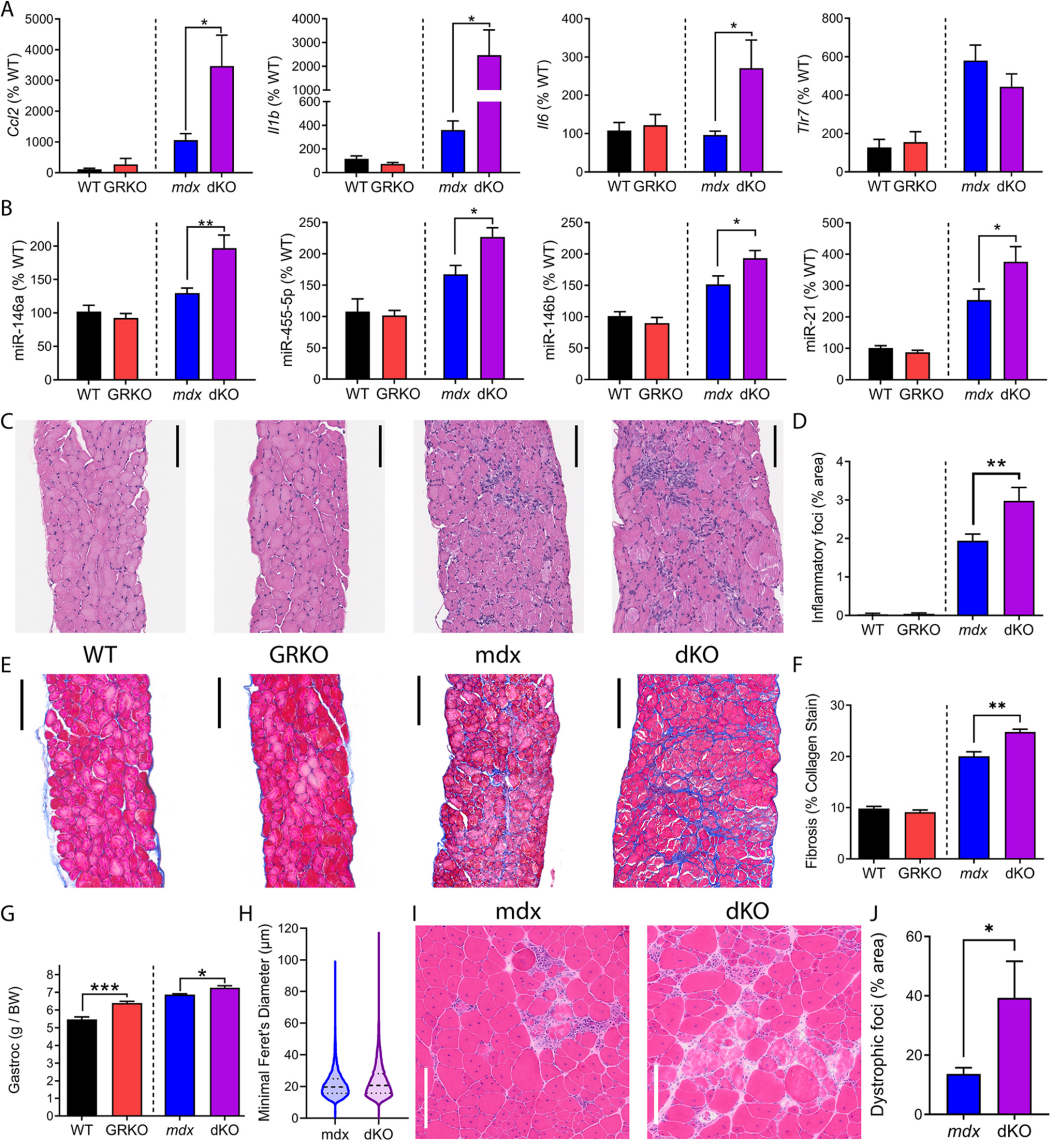
**The GR functions to limit inflammation and pathology in dystrophic muscle.** We assayed inflammatory gene expression, miRNA biomarkers and histopathology in mouse skeletal muscle. (A) Gene expression of gastrocnemius assayed via qRT-PCR showed that inflammatory gene expression significantly increased in dKO mice versus that in *mdx52* littermate controls. Consistent with chronic inflammation in muscular dystrophy, the levels of several of these transcripts were already increased in *mdx52* mice versus their levels in WT mice. (B) Levels of NF-κB-regulated miRNA biomarkers that are known to be elevated in inflammatory diseases were measured by miRNA-specific TaqMan assays. These miRNAs were increased in *mdx52* mice in comparison to WT mice, and significantly increased further in dKO versus *mdx52* littermates. (C,D) Consistent with gene expression data, H&E histopathology showed that *mdx52* mice presented with inflammation within dystrophic skeletal muscle (diaphragm), and that muscle-specific deletion of the GR further increased the amount of inflammatory infiltration within muscle. (E,F) Fibrosis was also visualized in diaphragm sections using Masson's trichrome staining, in which collagen stains as blue. Representative images are shown (E), and quantification shows that muscle-specific deletion of the GR resulted in a further increase of fibrosis in dystrophic *mdx52* muscle (F). Images are shown at equal magnification. (G) *mdx52* mice showed increased gastrocnemius muscle size relative to body weight (BW). Here, GRKO and dKO mice both showed increases relative to their littermate control genotypes at 3 months of age. (H) Violin plot of myofiber sizes as measured by the minimal Feret's diameter in dKO versus *mdx52* littermate controls. (I,J) Histopathology was assessed in H&E images. Representative images are shown (I) and quantification of the percentage of tissue consisting of dystrophic foci was performed (J). *n*=6 mice per group for A-G; *n*=3-4 mice per group for H-J. Data show mean±s.e.m. (significant outlier removed from A after ROUT test). *P≤0.05; **P≤0.01; mean±s.e.m.; unpaired two-tailed *t*-test of Cre-positive versus control littermates). Scale bars: 100 µm (C,E,I).

To determine whether the GR acts directly within muscle to impact inflammatory signaling, we assayed inflammatory gene expression, inflammatory microRNA (miRNA) biomarkers and histopathology in skeletal muscle from dKO mice versus *mdx52* littermates. We have previously found increased NF-κB-regulated gene expression in *mdx* muscles (Fiorillo et al., 2015; Heier et al., 2023). Here, we found that GR deletion further increased expression of the inflammatory genes *Ccl2*, *Il1b* and *Il6* (3- to 5-fold increase, *P*≤0.05) in dKO muscles (Fig. 5A). *Tlr7* showed no significant differences between *mdx52* and dKO. Similar results were found in diaphragm and quadriceps tissues, whereas no significant genotype effects were observed in non-muscle spleen and kidney tissues (Fig. S5).

In addition to messenger RNA transcripts, we also assayed inflammatory miRNAs that we have previously found to be elevated in *mdx* and regulated by NF-κB in diseases with chronic inflammation (Fiorillo et al., 2018; Batra et al., 2020; Heier et al., 2016, 2023; Kinder et al., 2020; Fiorillo et al., 2015). qRT-PCR showed elevated levels of *miR-146a* (*P*≤0.01), *miR-455-5p* (*P*≤0.05) and *miR-146b* (*P*≤0.05) in dKO muscle (Fig. 5B). Additionally, dKO mice showed increased levels of the NF-κB-regulated and pro-fibrotic miRNA *miR-21* (*P*≤0.05) compared to *mdx52* littermates (Fig. 5B).

To assess pathology in the muscle, we performed Hematoxylin and Eosin (H&E) staining as well as Masson's trichrome staining on diaphragm muscle tissue. We focused on the diaphragm as a skeletal muscle that is stressed relatively evenly among mice and typically shows a more advanced phenotype owing to its need for constant motion. Visually, H&E imaging revealed more apparent inflammation in dKO versus *mdx52* diaphragms (Fig. 5C). Quantitative analysis of *mdx52* diaphragms revealed the presence of inflammation in dystrophic muscle as expected, with a further significant increase of inflammation (*P*≤0.01) in dKO muscle versus in *mdx52* muscle (Fig. 5D). Examining trichrome-stained images, we saw a similar pattern in *mdx52* and dKO mice. Active fibrosis was visible in dystrophic muscle tissue, visualized by blue collagen staining (Fig. 5E), and this was significantly increased (*P*≤0.01) in dKO mice versus in *mdx52* mice (Fig. 5F).

Examining gastrocnemius as a muscle representative of transient or voluntary motion, we observed significant increases in gastrocnemius mass relative to body mass in both GRKO and dKO genotypes (*P*≤0.005 and *P*≤0.05, respectively) versus their littermate controls at 3 months of age (Fig. 5G). Raw mass values for gastrocnemius and body mass at the time of dissections did not show significant differences between genotypes (Fig. S6A,B). Using Sirius Red Fast Green (SRFG) staining to visualize collagen and laminin plus DAPI staining to image nuclei, we did not detect a significant increase in fibrosis or in the percentage of centrally nucleated fibers within gastrocnemius (Fig. S7A-C). Examination of myofiber size in histology sections showed a trend of larger fiber sizes by the measurement of minimal Feret's diameter, but there was no significant difference between the dKO and *mdx* genotypes (Fig. 5H). Quantification of pathology in gastrocnemius sections showed a significant increase in pathology, detected as the percentage of the tissue consisting of dystrophic foci within dKO mice versus that in *mdx52* littermate controls (Fig. 5I,J). Altogether, our data indicate that the physiological functions of GR play a protective role within dystrophic myofibers to help limit the degree of inflammation, histopathology and weakness phenotypes.

### The GR protects the heart from muscular dystrophy

**Fig. 6.**
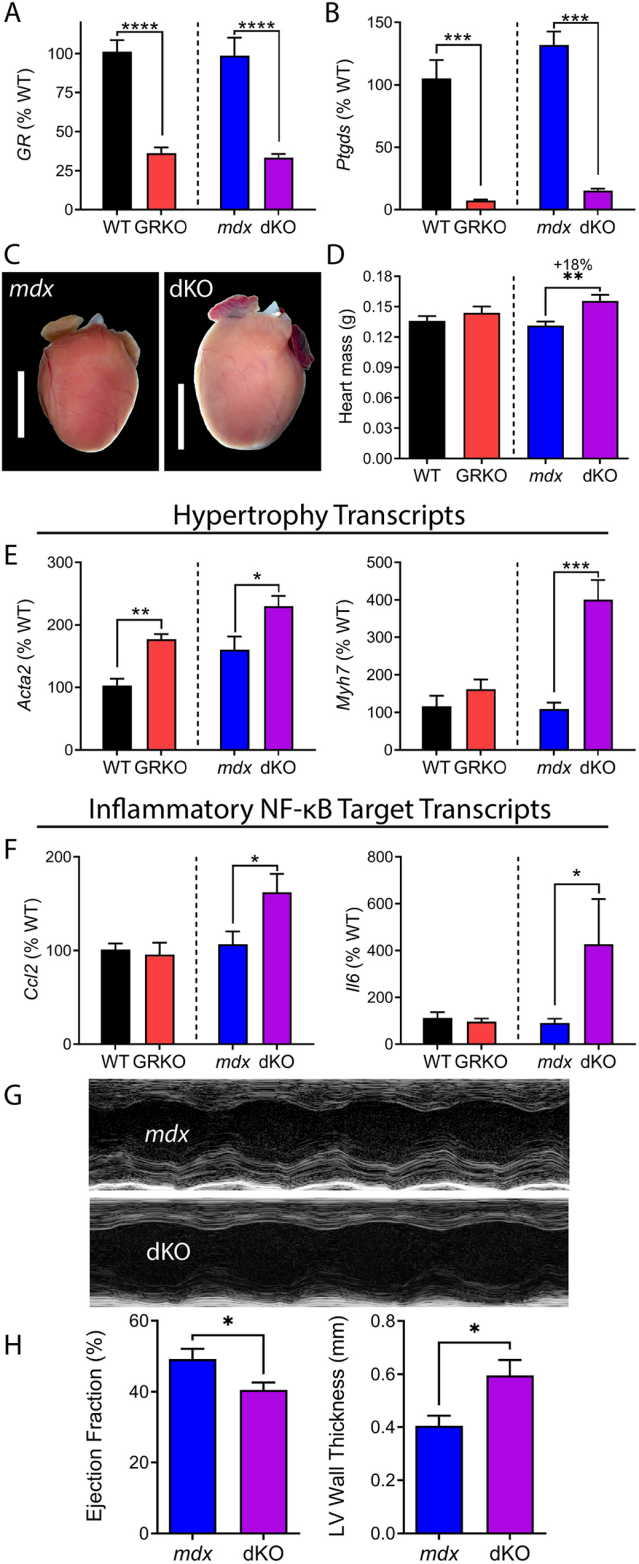
**Deletion of the GR worsens dystrophic cardiomyopathy.** (A) qRT-PCR confirmed knockout of the GR in the mouse heart. (B) Functional knockout of the GR was confirmed by assaying a known cardiomyocyte GR target gene (*Ptgds*). (C,D) Deletion of the GR in dKO versus *mdx52* mice resulted in visibly enlarged hearts (C) and a significant increase in heart mass (D). Scale bars: 4 mm. (E) The hypertrophy genes *Acta2* and *Myh7* were significantly upregulated in dKO hearts. (F) The inflammatory transcripts *Ccl2* and *Il6* were significantly upregulated specifically in dKO hearts. (G,H) Echocardiography of aged mice showed a worsening of dystrophic cardiomyopathy in 1-year-old dKO versus *mdx52* mice (*n*≥4 per group). (G) Representative M-mode images of the parasternal short axis. (H) Quantification of heart function via the ejection fraction demonstrates systolic dysfunction (left). Left ventricular (LV) wall thickness (right) measured at diastole showed an increase for dKO mice. *n*≥6 mice per group unless otherwise specified. Data show mean±s.e.m. (significant outlier removed from F after ROUT test). *P≤0.05; **P≤0.005; ***P≤0.0005; ****P≤0.0001; unpaired two-tailed *t*-test of Cre-positive versus control littermate genotypes.

We previously found dystrophin-null *mdx* hearts are specifically sensitized to pathology driven by mineralocorticoid receptor (MR, encoded by *Nr3c2*) signaling, and are negatively impacted by the dual GR and MR ligand prednisone (Heier et al., 2019, 2013). Here, we assayed dKO versus *mdx52* hearts to determine the impacts of GR knockout on dystrophic cardiomyopathy. The relevant physiological ligand that should be impacted in GR knockout mice is corticosterone, which is also capable of binding to both the GR and MR. Consistent with the results for skeletal muscle, efficient deletion of the GR was observed in heart muscle via qRT-PCR (Fig. 6A). Additionally, we detected clear functional loss of GR expression through a >90% decrease (*P*≤0.0001) in the expression of prostaglandin D2 synthase (*Ptgds*; Fig. 6B), which is known to be a cardiomyocyte GR-responsive gene (Tokudome et al., 2009). Upon dissection, we observed visibly enlarged hearts with significantly increased mass (∼18%, *P*<0.01) by 3 months of age in dKO mice versus those in *mdx52* littermate controls, whereas we did not observe these phenotypes in *mdx52* or single GR knockout (GRKO) mice (Fig. 6C,D).

To further dissect the molecular impacts of GR knockout on dystrophic hearts, we assayed genes associated with MR or *mdx* pathology. Consistent with their enlarged hearts, dKO mice showed significant increases in the expression of the cardiac hypertrophy genes *Acta2* (*P*≤0.05) and *Myh7* (*P*≤0.0005) (Fig. 6E). Interestingly, two patterns were observed: (1) increased gene expression was observed only in dKO hearts (*Myh7*) or (2) increased gene expression was observed in GRKO or *mdx52* hearts, and this was exacerbated in dKO hearts (*Acta1* and *Acta2*). We also examined gene expression linked to cytokine signaling, as *mdx* mice characteristically feature chronic inflammation. The inflammatory cytokines *Ccl2* and *Il6* showed significantly increased expression at 3 months of age in dKO mice versus in *mdx52* littermates (Fig. 6F). In histological sections, we did not detect a significant increase in fibrosis or dystrophic damage at this age (Fig. S7D-F). Taken together, data from 3-month-old hearts exhibit a pattern consistent with dKO mice showing earlier acceleration of transcriptional and mass phenotypes, at a time point that is normally pre-symptomatic for *mdx* cardiomyopathy.

Heart function was assessed at a symptomatic stage for *mdx* cardiomyopathy using echocardiography to determine whether the GR plays a protective role in maintaining heart function in dystrophic cardiomyopathy (Fig. 6G,H). Here, we assayed 1-year-old dKO mice versus *mdx52* littermate controls because it has previously been shown that *mdx* mice develop systolic dysfunction by this age (Spurney et al., 2009, 2008). It should be noted that two dKO mice and one *mdx52* mouse died from apparent sudden cardiac death during the echocardiography setup before data could be acquired; at least one of these may have been a stress response consistent with sudden cardiac death recently reported in the literature (Lindsay and Russell, 2023; Lindsay et al., 2021), as it occurred while transporting the mice to the echocardiography room prior to anesthesia. Quantification of heart function in live mice showed that endogenous GR deletion exacerbated left ventricular systolic dysfunction in *mdx52* mice, as dKO mice showed significant declines in the ejection fraction in comparison to that seen in *mdx52* littermates (−17.60%, *P*≤0.05, Fig. 6H). Additionally, we detected a significant increase in left ventricular wall thickness in dKO mice versus that in *mdx52* littermate controls. Taken together, these data indicate that the endogenous GR acts locally within the heart to maintain proper size, reduce inflammatory signaling, and maintain systolic function during the etiology of dystrophic cardiomyopathy in dystrophin-null mice.

## DISCUSSION

**Fig. 7.**
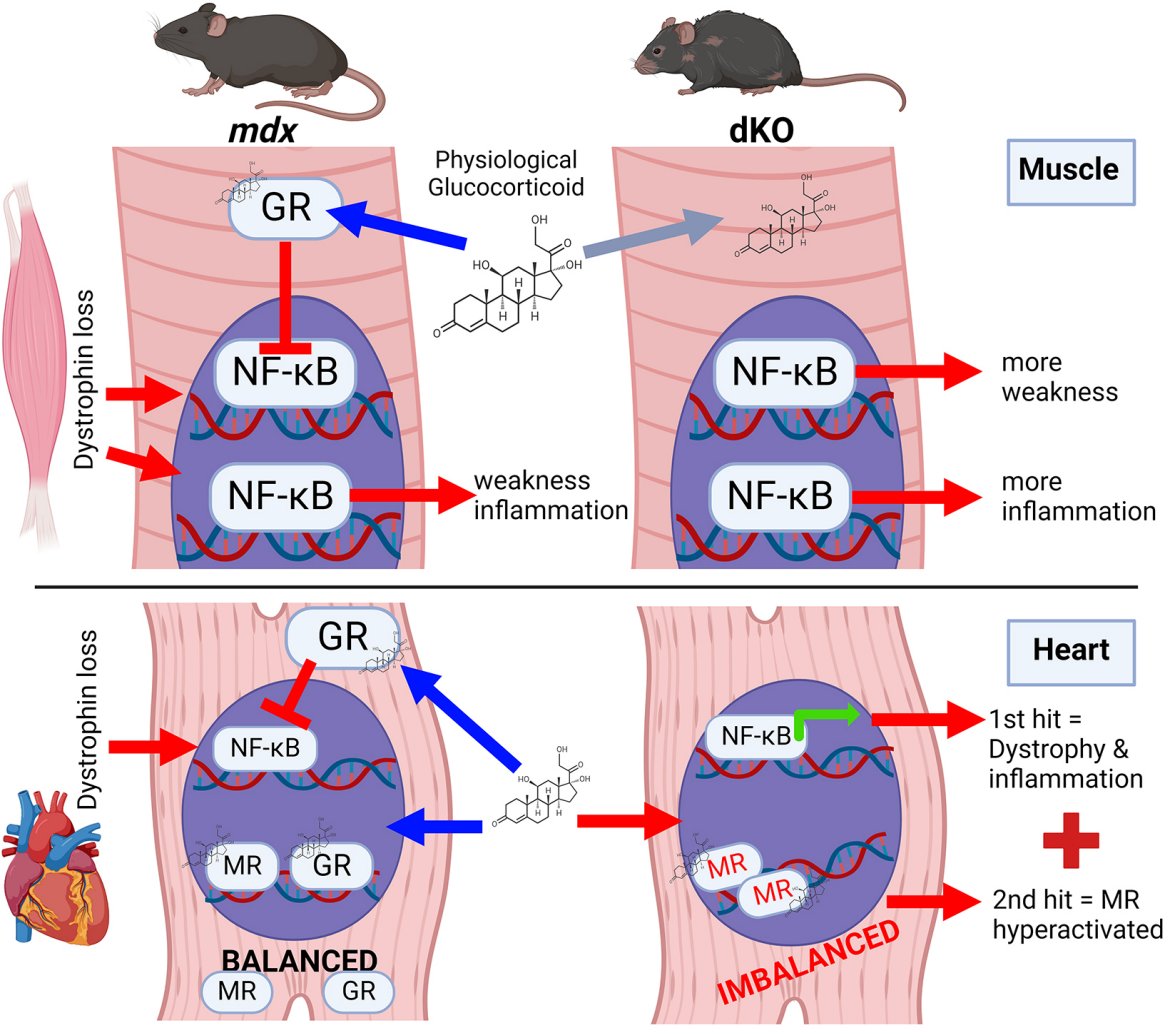
**Working model of GR protective roles in dystrophic myofibers and cardiomyocytes.** Dystrophin deficiency disrupts the stability of the sarcolemmal membrane, which leads to muscle disease characterized by progressive weakness and chronic inflammation. Elevated signaling by inflammatory transcription factors including NF-κB is present in patients with DMD at birth, years before symptom onset (Chen et al., 2005). By comparing *mdx52* and dKO mice in which the glucocorticoid receptor (GR) is deleted specifically within myofibers and cardiomyocytes, we found that the GR acts locally within these tissues to play a protective role. In this system, GR activity is mediated by the physiological ligand corticosterone in mice (cortisol in humans). In myofibers, deletion of the GR resulted in increased inflammatory signaling and a further progression of muscle weakness. In cardiomyocytes, deletion of the GR resulted in increased pathological gene expression, enlarged hearts and a further progression of cardiomyopathy with systolic dysfunction. Heart phenotypes are consistent with literature that establishes that imbalanced GR to MR levels worsen cardiomyopathy through overactivation of mineralocorticoid receptor (MR)-mediated pathology (Heier et al., 2019; Oakley et al., 2019). Without the GR to balance signaling or titrate away agonist dual-receptor ligands such as corticosterone or cortisol, MR hyperactivation can occur. Taken together, these findings indicate that physiological GR signaling acts locally to protect dystrophic muscle and heart during the natural etiology of disease in muscular dystrophy. Image created with BioRender.com.

The role of physiological glucocorticoids in muscular dystrophy has previously been unclear. Here we describe the generation and characterization of tissue-specific dKO mice generated using Cre/LoxP recombination to induce myofiber and cardiomyocyte-specific deletion of the GR in DMD model mice. Knockout of the GR in healthy muscle does not have a clear impact other than increased gastrocnemius mass relative to body weight, whereas clear effects on *mdx* muscle suggest a dystrophy-dependent effect of GR deletion. dKO mice showed a further progression or acceleration of dystrophic weakness, inflammation, muscle and cardiomyopathy phenotypes versus those seen in control *mdx52* littermates. These data show that physiological GR signaling plays a protective role by acting locally within myofibers and cardiomyocytes to protect these tissues against the dystrophic disease process (summarized in Fig. 7).

Physiological glucocorticoids are linked to muscle atrophy and inflammatory muscle wasting in several diseases. In mice, the physiological glucocorticoid is corticosterone, whereas in humans, it is cortisol. Previous rodent studies have shown that excess corticosterone is associated with muscle wasting in models of diabetes (Hu et al., 2009), sepsis (Voisin et al., 1996), starvation (Wing et al., 1995) and metabolic acidosis (May et al., 1986). In a direct study on how the GR influences inflammation in muscle, Braun et al. (2013) found that muscle-specific GR deletion causes >70% improvement in muscle atrophy caused by tumor growth or by endotoxin (lipopolysaccharide) administration. This indicates that in some inflammatory disease states, physiological GR signaling is required for and participates in muscle wasting. In such diseases, a viable combination therapy could be GR depletion in combination with anti-inflammatory treatment. Interestingly, prednisone possesses both of these properties; chronic treatment with pharmacological glucocorticoids can cause downregulation of the GR at the mRNA and protein level (Silva et al., 1994; Shimojo et al., 1995; Andreae et al., 2001) as well as downregulation of physiological GR ligands through adrenal suppression as seen in patients with DMD (Hathout et al., 2016). From those collective studies, an alternative hypothesis going into our current study was that muscle-specific GR deletion would improve *mdx* disease. However, here we found the opposite. In *mdx52* mice, deletion of the GR exacerbated disease in the heart and skeletal muscle. This indicates that the GR and its physiological ligands provide a protective function against the dystrophic disease processes in DMD, in contrast to its role in certain other disease states featuring muscle atrophy or inflammatory muscle wasting.

Pharmacological glucocorticoids can also cause muscle weakness and atrophy in other disease states; however, they improve motor function, mortality and cardiac outcomes in patients with DMD, for which they are the standard of care. This paradox has historically led to debate and a lack of clarity over the molecular actions of glucocorticoids in muscular dystrophy, with some proposing that the benefits of pharmacological application come from impaired growth or immunosuppression (Grounds and Shavlakadze, 2011; Finkel et al., 2021; Griggs et al., 1993; Grounds and Torrisi, 2004; Hammers et al., 2016). New models show a consistent paradox in mice, but these are now allowing us to clarify these issues. In examining the cellular autonomy of glucocorticoid-driven muscle wasting, Watson et al. (2012) showed that muscle atrophy is efficiently induced in WT mice by dexamethasone, whereas muscle-specific deletion of the GR prevents this effect. The development of the dissociative steroid vamorolone in *mdx* mice shows that a GR ligand can improve muscle function and pathology in muscular dystrophy without prednisone-like side effects of impaired growth or immunosuppression (Heier et al., 2013, 2019); consistent results are now being seen in human trials, in which vamorolone improves DMD outcomes with greatly reduced side effects to growth and bone (Hoffman et al., 2019; Mah et al., 2022; Smith et al., 2020; Dang et al., 2024). Here, we find that physiological GR signaling acts cell-autonomously to protect dystrophin-null muscle, which is more consistent with the protective role of the GR as a drug target in DMD.

We hypothesize that the GR acts locally to inhibit inflammatory programs that are inappropriately upregulated within dystrophic myofibers themselves, as opposed to solely within immune or myeloid tissue types. This is consistent with the results here showing significantly worse phenotypes for *mdx52* mice that have a myofiber-specific GR knockout. In the past, alternative attempts to develop anti-inflammatory drugs for DMD have focused on strategies that target specific inflammatory signaling pathways in myeloid cells or cause general immunosuppression. However, despite promising preliminary data in animal models, so far, these alternative strategies have not shown efficacy in clinical trials. These include the NF-κB inhibitor edasalonexent, which recently failed to show significant efficacy in phase 3 trials (Finkel et al., 2021), the anti-TNF biologic infliximab and the immunosuppressive drug azathioprine (Griggs et al., 1993; Kissel et al., 1993). In contrast, the dissociative steroid vamorolone shares the GR with prednisone as its drug target, and both show significant improvements of DMD in patients (Hoffman et al., 2019; Mah et al., 2022; Smith et al., 2020). This suggests that the efficacy of corticosteroid drugs comes from properties specifically related to the targeting of GR within myofibers to inhibit inflammation. Moving forward, future investigations with this dKO model should define cell-autonomous roles of the GR in muscle, satellite cells and immune cell types in disease as well as in the pharmacological treatment of muscular dystrophy.

An alternative hypothesis is that GR ablation in normal tissues has a minimal effect, and this effect is exacerbated on a dystrophic background. Further insights into the interplay between GR, NF-κB and MR signaling may clarify the separability of these pathways. Regarding the GR and NF-κB, the GR can inhibit NF-κB-driven transcription even when NF-κB is bound to the promoters of its traditional gene targets such as *IL6* (de Bosscher et al., 1997, 2000). Crystal structure and solution NMR data have established that the GR can bind to NF-κB target gene promoters through cryptic GR-binding sites found between DNA-bound NF-κB subunits (Hudson et al., 2018). These data suggest that the GR directly represses NF-κB target promoters by interfering with transcriptional machinery or by changing cofactor interactions. Removal of the GR thus may directly result in the increased inflammatory signaling we see via unchecked NF-κB at inflammatory gene promoters. However, one alternative explanation to the increased inflammation in dKO mice could be that the GR itself exerts a myoprotective function and its ablation leads to increased myofiber death in a sensitized dystrophic background, with greater inflammation then resulting downstream of this function. Regarding the MR, recently, MR antagonists have been found to reduce cytokine expression in *mdx* quadriceps muscle and myeloid cells (Howard et al., 2022). Thus, consistent with our observations and model in dystrophic hearts, the pathological effects of GR ablation in skeletal muscle could conversely be exacerbated in dKO mice by imbalanced MR activation. Moving forward, it will be interesting to determine the impacts of MR antagonists in GR dKO mice, of myeloid GR knockout, or of further molecular dissection of promoter regulation.

Although our focus was on the muscle, several observations were made in kidney tissue here. For one, the Dp71 isoform of dystrophin was found to be expressed at relatively high levels in the kidney, consistent with past findings from other groups (Duan et al., 2021; Haenggi et al., 2005; Loh et al., 2000). Examining GR protein levels, we observed increased variability in the kidney along with potential compensatory upregulation in kidney tissue from muscle GR knockout genotypes. If such upregulation was detrimental to renal function, this could add to impacts on the muscle as chronic kidney disease can be associated with reduced muscle mass and function. This would be consistent with the reduced muscle function we saw in muscle-specific dKO mice, although we did not see a reduction in muscle mass.

Examining our results in the heart, several interesting connections or comparisons can be made between our study and others examining cardiomyocyte-specific GR knockout mice. One is that we saw no clear impact of GR deletion on mouse survival and minimal effects on the heart in single GR knockout mice. This is in contrast to studies that show that Cre-mediated GR deletion in cardiomyocytes results in cardiomyopathy and premature mortality of mice beginning by 6 months of age (Oakley et al., 2019, 2013). A likely explanation for this difference comes from the fact that these prior studies used a particular transgenic line that utilizes an α-myosin heavy chain (αMHC) promoter to drive expression of Cre recombinase in cardiomyocytes. Prolonged expression of Cre recombinase from this specific promoter is now known to be cardiotoxic (Pugach et al., 2015; Agah et al., 1997). Cre recombinase is highly expressed from six tandem repeats inserted as a transgene in the commonly used αMHC-Cre line. Prolonged overexpression of Cre in these mice results in cardiotoxicity, heart dysfunction, active DNA damage responses and off-target deletion of up to 55 endogenous heart-expressed genes via cryptic LoxP sites in the mouse genome (Gillet et al., 2019; Pugach et al., 2015). Cardiomyocyte-specific Cre expression via other promoters or transgenic lines does not show the same cardiotoxicity; this has been found both for a troponin T (TNT) promoter and for a transgenic line that also uses an αMHC promoter but shows reduced expression levels (Abel et al., 1999; Pugach et al., 2015; Gillet et al., 2019). Despite these findings, authors of the GR cardiomyocyte knockout paper (Oakley et al., 2019) included appropriate controls and most effects should ultimately result from glucocorticoid signaling; however, it is likely that several phenotypes in this study, such as survival, may manifest because the αMHC-Cre transgene induces extraneous off-target deletions that synergistically predispose mice to cardiotoxicity.

Glucocorticoids are an important stress signal that can bind both the GR and MR in heart tissue, and previous studies show that a dystrophin-null genotype acts as a ‘second hit’ to predispose hearts to damage via MR activation (Heier et al., 2013, 2019). Specifically, aldosterone causes *mdx* hearts to become enlarged and develop systolic dysfunction, whereas MR inhibitors prevent this effect. This is consistent with other diseases in which MR activation on its own is benign; however, introduction of a second hit such as inflammation, infarction, oxidative stress or a high salt diet leads to cardiomyopathy (Brilla and Weber, 1992; Sun et al., 2002; di Zhang et al., 2008; He et al., 2011; Ruhs et al., 2012; Fraccarollo et al., 2011). Our current results are consistent with this model. Here, GR deletion frees the physiological glucocorticoid corticosterone to bind at higher levels to the MR, leading to MR overactivation. In dKO mice, a dystrophin-null background acts as a second hit, leading to enlarged hearts with cardiac dysfunction, consistent with previous studies of aldosterone exposure in *mdx* mice (Heier et al., 2019). Interestingly, in the cardiomyocyte-specific single GR knockout studies discussed above (Oakley et al., 2019, 2013), the αMHC-Cre transgene has been found to also cause off-target deletion of dystrophin via cryptic LoxP sites in mice, whereas other Cre-expressing mice (such as TNT-Cre) avoid this (Gillet et al., 2019). This means that dystrophin deletion, along with other off-target gene deletions, is also present as a second hit in this model; the authors indeed note a decrease in dystrophin RNA levels but theorize that it was a secondary effect of cardiomyopathy, then find that a dual deletion of the MR prevented many of the heart phenotypes in GR-null mice (Oakley et al., 2019). Together, our data and previous reports are consistent with a two-hit model in which MR activation acts synergistically with dystrophin deletion to accelerate or drive dystrophic cardiomyopathy (Fig. 7).

Novel compounds that selectively activate anti-inflammatory GR properties in myofibers and antagonize MR signaling in cardiomyocytes have the potential to provide improved therapeutics for muscular dystrophy. Vamorolone is a new dual GR/MR ligand that has both of these properties (Heier et al., 2019) and was recently approved by the US Food and Drug Administration (FDA), European Medicines Agency (EMA) and the Medicines and Healthcare products Regulatory Agency (MHRA) in the UK, after completing DMD clinical trials in which it was tested in comparison to prednisone (Hoffman et al., 2019; Mah et al., 2022; Smith et al., 2020). Originally developed in *mdx* mice, vamorolone increases the strength of patients with DMD while showing greatly reduced impacts to growth and bone turnover, in comparison to the effects of prednisone (Guglieri et al., 2022; Dang et al., 2024). We found that it increases *mdx* mouse strength without impaired growth or immunosuppression (Heier et al., 2013), suggesting that these properties are dispensable for DMD efficacy. Although other anti-inflammatories have failed clinical trials (Finkel et al., 2021), our results here, along with the consistent efficacy of prednisone, deflazacort and vamorolone, indicate that the specific mechanism by which the GR ligands inhibit inflammation in myofibers may be an essential driver of efficacy in DMD. Future studies will help to further dissect precise pathways, relevant tissues and/or alternative drug targets for the development of even more selective therapeutics.

One limitation of the current study is that the GR is deleted before phenotypic onset, whereas in patients, glucocorticoids are typically given after the initial manifestation of symptoms. In the future, it would be interesting to see the effects of deleting the GR from myofibers after phenotypic onset in DMD model mice. Additionally, clinical and genotypic heterogeneity could lead to differential responses to glucocorticoids in patients, whereas here, we exclusively tested GR deletion on the *mdx52* mouse with a C57BL/6 background. Towards investigating this, it would be interesting to examine the impacts of GR deletion in differing dystrophin-deficient models or genetic backgrounds. For example, the *bmx* mouse model of Becker muscular dystrophy would provide a backdrop of partially functional Becker-like dystrophin isoforms (Heier et al., 2023; McCormack et al., 2023) as opposed to dystrophin-null *mdx52* mice. Additionally, the D2.*mdx* mouse presents a dystrophin-null model that has a more severe phenotype with increased fibrosis and cardiac dysfunction (Coley et al., 2016; Hammers et al., 2020).

In conclusion, our characterization of dKO mice demonstrates that physiological GR signaling plays a protective role in muscular dystrophy. It acts locally in myofibers to dampen or limit the progression of weakness and inflammation. Additionally, it acts locally in cardiomyocytes to reduce inflammatory signaling and to balance MR signaling, which is a source of pathology that dystrophin-deficient hearts are specifically sensitized to. Moving forward, this model will be useful to define the mechanisms of pharmacological GR ligands and to dissect cell-autonomous roles of the GR in muscle versus in other tissues such as satellite cells or macrophages. By dissecting the molecular mechanisms of these pathways, we can develop improved treatments, identify new drug targets and translate treatments to new patient populations.

## MATERIALS AND METHODS

### Animal care and maintenance

All animal work was conducted with the species *Mus musculus* according to relevant institutional, national and international guidelines, with adherence to standards of the National Institutes of Health Guide for the Care and Use of Laboratory Animals. All *mdx52* experiments were conducted according to the protocols approved by the Institutional Animal Care and Use Committee of Children's National Hospital. Animals were maintained in a controlled mouse facility with a 12 h:12 h light:dark photoperiod, fed *ad libitum*, and monitored daily for health. Phenotyping and histopathology studies were masked to genotype. Exclusively male mice were used for these experiments because DMD is an X-linked disease primarily affecting males.

### Creation of tissue-specific single- and double-knockout mouse strains

For conditional GR deletion, a strain of mice featuring a LoxP-flanked exon 2 allele of the GR gene (*GR^flox^*) was purchased from The Jackson Laboratory (strain 012914; *B6.129S6-Nr3c1tm2.1Ljm/J*). For tissue-specific expression of Cre recombinase, a strain of mice expressing Cre from a creatine kinase, muscle (Ckm)-type promoter (*Ckmm-Cre*) was purchased from The Jackson Laboratory (strain 006475). C57/BL6-*mdx*Δ*52* mice (*mdx52*) contain a deletion of exon 52 of the dystrophin gene (*Dmd*), resulting in the absence of full-length dystrophin, and were originally provided as a gift by Dr Shin'ichi Takeda (National Center of Neurology and Psychiatry, Kodaira, Japan). All strains of mice were obtained and maintained on a C57/BL6 background. Genotyping was performed using assays established by TransnetYX. All strains were maintained in-house at Children's National Hospital.

To develop single muscle-specific GRKO mice, the *GR^flox^* and *Ckmm-Cre* lines were crossed to produce Cre-positive mice heterozygous for *GR^flox^*. These mice were then bred to obtain single-knockout breeder pairs consisting of mice that were homozygous for *GR^flox^* alleles, and either hemizygous for *Ckmm-Cre* or lacking Cre. These breeders were then used to generate litters consisting of half Cre-positive GR knockout mice (abbreviated here as GRKO; genotype *Dmd^+/Y^*:*GR^flox/flox^*:*Ckmm-Cre^+/−^*) and half Cre-negative and GR-positive control littermates (abbreviated here as WT; genotype *Dmd^+/Y^*:*GR^flox/flox^*:*Ckmm-Cre^−/−^*).

In parallel, *mdx52* mice were crossed to *GR^flox/flox^* mice to obtain mice with a dystrophic *mdx52* allele and heterozygous for *GR^flox^*. Subsequent crosses were performed to produce a breeding line that consisted of *mdx52* mice also homozygous for *GR^flox^*. To produce dKO mice, we set up test crosses composed of female *mdx^−/−^:GR^flox/flox^* mice and male *GR^flox/flox^:Cre^+/−^* hemizygotes. All male offspring from these crosses were dystrophin-null *mdx52* due to X inheritance from the mother, and all offspring were homozygous for *GR^flox^*. Half of the mice from these test crosses were Cre-positive (abbreviated here as dKO; *Dmd^−/Y^*:*GR^flox/flox^*:*Ckmm-Cre^+/−^*), whereas the other half were Cre-negative control littermates (abbreviated here as *mdx52*; genotype *Dmd^−/Y^*:*GR^flox/flox^*:*Ckmm-Cre^−/−^*).

### Protein analysis via capillary western immunoassay

Muscles were flash frozen in liquid nitrogen immediately upon dissection at 6 months of age. Frozen tissues were ground using a liquid nitrogen-cooled grinder, then lysed for protein using RIPA buffer (Thermo Fisher Scientific, 89900) containing cOmplete ULTRA protease inhibitors (Millipore Sigma, 05892970001). Capillary western immunoassay was performed using either 12-230 kDa (for GR and GAPDH) or 66-440 kDa (for dystrophin and vinculin) separation modules (ProteinSimple) according to the manufacturer's instructions. The following antibodies were used: anti-GR (D8H2) XP (Cell Signaling Technology, 3660, 1:15), anti-GAPDH (14C10) (Cell Signaling Technology, 2118, 1:300), anti-dystrophin C-terminus (Abcam, ab15277, 1:15), anti-vinculin (Abcam, ab130007, 1:100) and anti-rabbit secondary HRP antibody (ProteinSimple, 042-206, 10 μl per well). The Dp71 isoform was also visualized using the antibody against the dystrophin C-terminus, with the detected isoform running just above 66 kDa for the molecular mass ladder. Full capillary western immunoassay images are shown in Figs S8 and S9.

### Immunofluorescence microscopy analysis

Tissues from 3-month-old mice were dissected, mounted on cork with tragacanth gum, frozen in liquid nitrogen-cooled isopentane, and sectioned at 8 µm using a CM1950 cryostat (Leica Biosystems) onto slides. Muscle sections were fixed in ice-cold acetone for 10 min. Slides were then washed, blocked for 1 h (1× PBST with 0.1% Triton X-100, 1% bovine serum albumin, 10% goat serum and 10% horse serum), washed three times, then exposed to primary antibodies overnight at 4°C. The secondary antibodies were applied for 1 h at room temperature. The primary antibodies used were anti-laminin-2 (1:100, Sigma-Aldrich, L0663, clone 4H8-2 rat monoclonal antibody) and anti-GR (1:2000, Cell Signaling Technology, 3660, clone D8H2 rabbit monoclonal antibody). The secondary antibodies used were Alexa Fluor 568 goat anti-rabbit IgG (1:400, Thermo Fisher Scientific, A-11036), Alexa Fluor 488 donkey anti-rat IgG (1:400, Thermo Fisher Scientific, A-21208) and Alexa Fluor 647 goat anti-rat IgG (1:400, Thermo Fisher Scientific, A-21247). Coverslips were mounted with Prolong Gold mounting medium (Thermo Fisher Scientific, P36931) with DAPI. Sections were imaged at 20× magnification using a VS120 microscope (Olympus).

GR presence or absence was assayed in skeletal muscle and heart sections. Myofibers or cardiomyocytes were considered positive for GR if they contained nuclei that stained positive (red) for GR, the GR colocalized with DAPI (blue) and the nuclei was internal to muscle membranes stained with laminin (green). To quantify the percentage of GR-positive myofibers or cardiomyocytes, we counted all fibers present in representative images. Here, four representative 10×images per gastrocnemius section and three representative 30× images per heart section were selected by visualizing laminin and DAPI, and randomly dispersed areas across the section considered to consist primarily of myofibers and not primarily active areas of necrosis and inflammation were selected (*n*=3-4 mice per group). In total, at least 350 myofibers per muscle section and at least 97 cardiomyocytes per heart section were quantified.

Myofiber size was assayed by measuring the minimum Feret's diameter using the MuscleJ macro for FIJI (Mayeuf-Louchart et al., 2018) (*n*=3-4). Centrally nucleated fibers were counted manually. Total muscle section area was determined using ImageJ.

### Serum ELISAs and reporter assay

Blood was collected from mice via the retro-orbital route and allowed to clot at room temperature for 60 min. Samples were obtained from 6-month-old mice. The serum was collected after centrifugation at 4°C and stored at −80°C. Circulating levels of the cytokine IL-6 (Abcam, ab222503) and the physiological steroid corticosterone (Arbor Assays, K014-H1) were assayed by ELISA according to the manufacturer's instructions.

Steroid inhibition of inflammatory NF-κB was assayed through an *in vitro* reporter system consisting of an NF-κB luciferase reporter stably expressed within a HeLa cell line and tested within the first few passages of purchase from the vendor (Signosis, SL-0001-NP). Cells were plated overnight at 10,000 cells per well in a 96-well plate. Reporter cells were pre-treated for 1 h with vehicle (DMSO) or drug, either 10, 100 or 1000 ng/ml corticosterone (Sigma-Aldrich, 27840) or 1000 ng/ml prednisolone (Sigma-Aldrich, P6004). Inflammatory signaling was induced with 10 ng/ml TNF-α (Gibco, PHC3015). After 4 h of stimulation, luciferase activity was quantified using the Dual-Glo Luciferase Assay System (Promega, E2920).

### Phenotyping assays

Forelimb and hindlimb grip strength was assessed in mice at 5 weeks of age using a grip strength meter (Columbus Instruments) daily for five consecutive days according to Treat NMD protocols (DMD_M.2.2.001; https://www.treat-nmd.org/resources-and-support/sop-library/mdx-mouse-dmd/), with data interpreted as averaged maximum daily values. Two-limb wire-hang and four-limb grid-hang tests were performed at 7 and 8 weeks of age, respectively, in accordance with Treat NMD protocols (DMD_M.2.1.005; https://www.treat-nmd.org/resources-and-support/sop-library/mdx-mouse-dmd/). For the two-limb wire-hang assay, a wire hanger was suspended ∼35 cm above a cage with soft bedding. Mice were hung using only their forelimbs; however, they were allowed to swing and hang with all four limbs if able. Hang time was recorded, with 10 min used as a cutoff. For four-limb grid-hang tests, the same parameters were used, but mice were instead hung upside down from a handmade box covered in wire mesh (1×1 cm grid). *n*≥12 mice per group were used for analysis.

No apparent impacts of genotype were observed on the survival of younger mice. To determine the full life expectancy for mice of each genotype, mice reaching 1 year of age were placed into a survival study and observed regularly until they met death endpoints. The full natural lifespan for each genotype was observed to the maximal length of 36 months (*n*≥17 per genotype).

### Gene expression

qRT-PCR was performed as previously reported (McCormack et al., 2023). Frozen tissues from mice dissected at 3 months of age were ground using a liquid nitrogen-cooled grinder, then homogenized in 1 ml TRIzol (Life Technologies) using a TissueRupter II homogenizer (QIAGEN). To test mRNA expression, RNA was isolated from TRIzol lysates. cDNA was synthesized from 1000 ng total RNA using a High-Capacity Reverse Transcription Kit (Thermo Fisher Scientific, 4368813) and multiplexed RT primers. qRT-PCR was performed using TaqMan Fast Advanced Master Mix (Thermo Fisher Scientific, 4444557) and TaqMan probes (Thermo Fisher Scientific). miRNAs were quantified using individual TaqMan assays on a QuantStudio 7 real-time PCR machine (Applied Biosystems). The assay IDs were: Cre recombinase, Mr00635245_cn; GR (*Nr3c1*), Mm00433832_m1; dystrophin at its 3′ end, 00464531_m1; *Ccl2*, Mm00441242_m1; *Il1b*, Mm00434228_m1; *Il6*, Mm00446190_m1; *Tlr7*, Mm00446590_m1; *Ptgds*, Mm01330613_m1; *Acta2*, Mm01546133_m1; *Acta1*, Mm00808218_g1; *Myh7*, Mm00600555_m1; *Hprt*, Mm01545399_m1; and 18S rRNA, Mm03928990_g1. qRT-PCR data were normalized to the geometric mean of the levels of the control *Hprt* gene and 18S rRNA.

miRNAs were quantified using individual TaqMan assays on the QuantStudio 7 real-time PCR machine as previously described (Fiorillo et al., 2015). The assay IDs were: *miR-146a*, 000468; *miR-146b*, 001097; *miR-455-5p*, 001280; *miR-21*, 000397; *sno202*, 001232; and *U87*, 001712. qRT-PCR data were normalized to the geometric mean of the levels of the *sno202* and *U87* control genes.

### Pathology

Tissue weights were obtained during dissections at 3 months of age. Diaphragms were fixed in 10% formalin, embedded in paraffin, cross-sectioned, and stained with either H&E to assess tissue pathology or Masson's trichrome to image collagen deposition. Infiltration of diaphragm muscle by inflammatory cells was analyzed in H&E-stained diaphragms as in Heier et al. (2013) and expressed as a percentage of total tissue area. Fibrosis of skeletal muscle was analyzed in trichrome-stained images using ImageJ to separate images by color using the Lab Stacks function, followed by using the threshold function to measure the percentage of area with blue staining (collagen) in relation to the total tissue area. For each sample, full tissue sections were imaged using a VS120 scanning microscope, then the areas of inflammation, fibrosis and/or total tissue area were measured for these full sections by scorers who were unaware of sample identity using ImageJ.

Heart and gastrocnemius muscles were dissected, mounted on cork with tragacanth gum, frozen in liquid nitrogen-cooled isopentane, and sectioned at 8 µm onto slides. Sections were stained with either H&E to assess tissue pathology or SRFG stain to image collagen deposition, with assessments following protocols similar to those described above. H&E images were assessed for pathology by quantifying areas of inflammation and necrosis (dystrophic foci). Fibrotic staining was assessed in SRFG images via ImageJ by thresholding to measure the percentage of area with red staining (collagen) in relation to the total tissue area.

### Echocardiography

Echocardiography was performed as previously described (Heier et al., 2019). Briefly, aged (1-year-old) *mdx52* and dKO mice (*n*=4-5) were assayed using a Vevo 3100 micro-ultrasound imaging system (VisualSonics). Images were acquired via high-resolution electrocardiogram-gated kilohertz visualization as well as via M-mode imaging of the parasternal long axis and the parasternal short axis. Image analysis, measurement of left ventricular wall thickness at diastole and calculation of cardiac ejection fractions was performed using Vevo software.

### Statistical analysis

Statistical analyses were performed for all assays by direct comparison of each GR knockout genotype to its littermate controls via unpaired two-tailed *t*-test; specifically, GRKO mice were compared to WT littermate controls, and dKO mice were compared to *mdx52* littermate controls. Phenotyping and histopathology assays were performed with the genotype masked. Tissues were randomly selected to obtain a sample size, typically *n*=6 or as denoted in the figure legends, for molecular and histology assays. Data were tested for outlier values using a ROUT test (GraphPad Prism 10.0.1) where denoted in figure legends. Values of *P*≤0.05 were regarded as statistically significant. For all graphs, data are presented as mean±standard error of the mean (s.e.m.).

## Supplementary Material

10.1242/dmm.050397_sup1 Supplementary information
