# Supplementary material for: The glucocorticoid receptor acts locally to protect dystrophic muscle and heart during disease
Source: Dis Model Mech. 2024 May 21;17(5):dmm050397. doi: 10.1242/dmm.050397 (PMC11139035; doi:10.1242/dmm.050397)
Supplement: Supplementary information [file dmm-17-050397-s1.pdf]

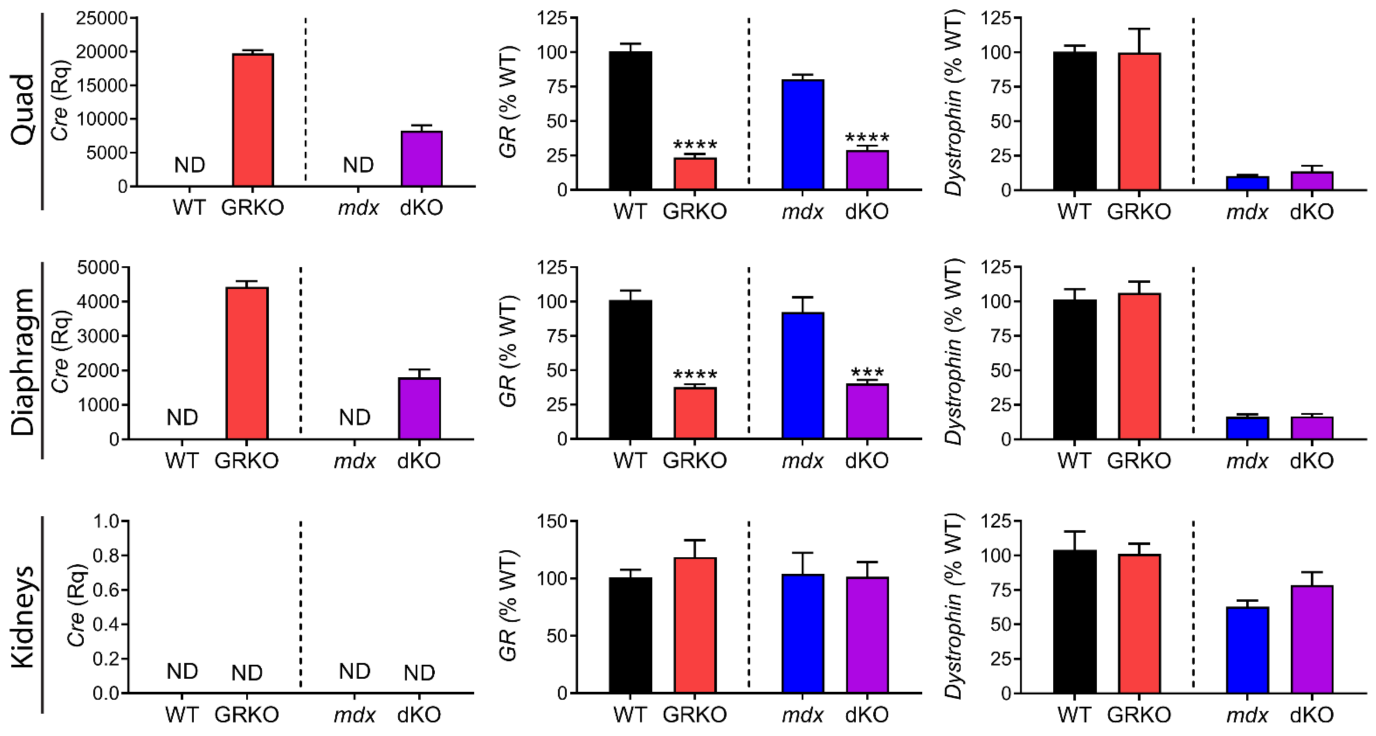

Fig. S1. Extended validation of tissue-specific double knockout mice. qPCR of muscle (quadriceps and diaphragm) and non-muscle (kidney) tissues from three-month old mice confirms expression of Cre, knockdown of GR, and absence of dystrophin specifically in muscle tissue. *n*=6 per group. Data show mean±s.e.m. ****P*≤0.0005, *****P*≤0.0001; unpaired two-tailed *t*-test of Cre-positive vs. littermate control genotypes.

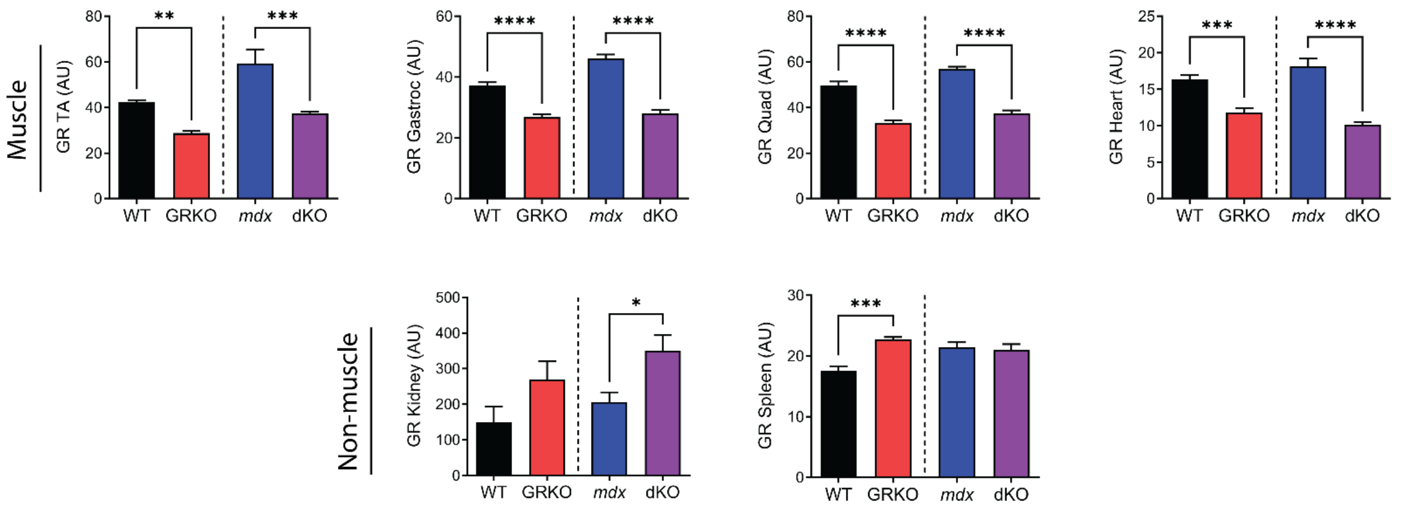

Fig. S2. Quantification of capillary western protein signal. Quantification of capillary signals is provided for the tissues assayed in muscle (TA, Gastroc, Quad, Heart) and non-muscle tissues from WT, GRKO, mdx52 and dKO genotypes. *n*=6 per group. Data show mean±s.e.m. ***P*<0.005, ****P*<0.0005, *****P*<0.0001; unpaired two-tailed *t*-test of Cre-positive vs. control littermates.

Disease Models & Mechanisms • Supplementary information

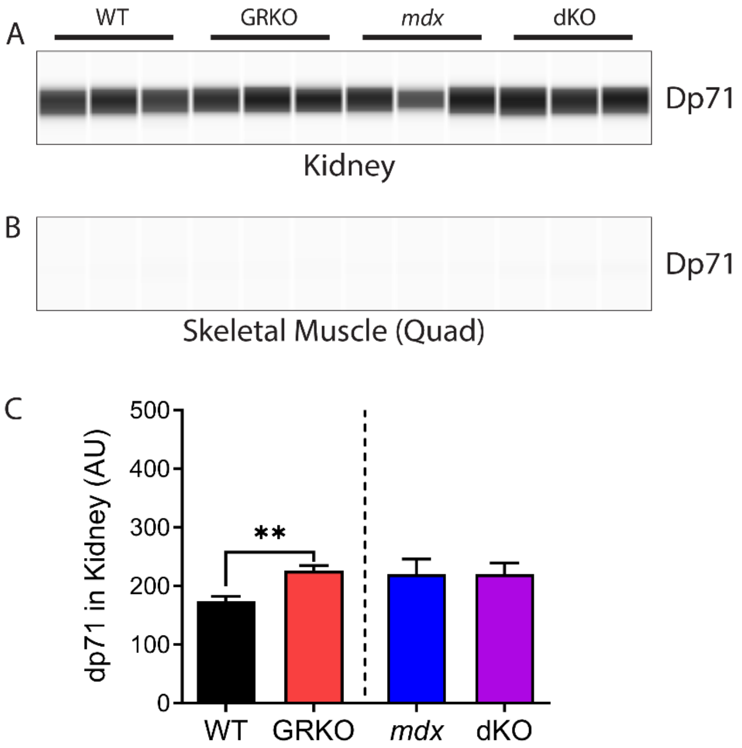

Fig. S3. Detection of Dp71 isoform of dystrophin in kidney. The Dp71 isoform of dystrophin is expressed in non-muscle tissues. Here Dp71 presence was assayed via Wes immunoassay with dystrophin antibody detection just above the 66 kDa mark of the molecular weight ladder. Representative images from the corresponding dystrophin Wes capillary electrophoresis experiments in Fig. 2 are provided for (A) Kidney, and (B) Quadriceps skeletal muscle at the same molecular weight position. (C) Quantification of immunoassay signal in Kidney. *n*=6 per group. ***P*<0.01; unpaired two-tailed *t*-test of Cre-positive vs. control littermates.

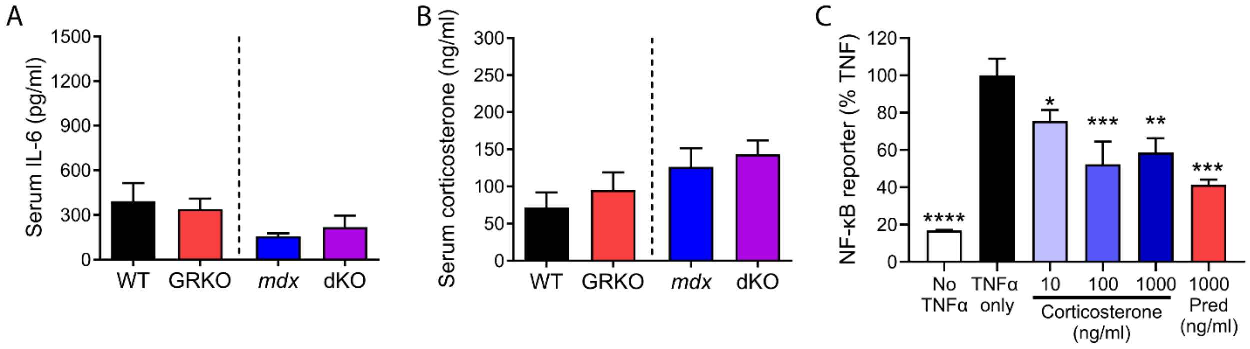

Fig. S4. Analysis of serum IL-6 and corticosterone levels. (A) ELISA of IL-6 levels in mouse serum samples. (B) ELISA of corticosterone levels in mouse serum samples. (C) *In vitro* experiment shows inhibition of an NF-κB luciferase reporter at concentrations of corticosterone found to be within physiological levels of mouse serum samples. *n*=6 per group (A-B); *n*=4 per group (C). **P*<0.05, ***P*<0.005, ****P*<0.0005, *****P*<0.0001; ANOVA with Holm-Sidak post-hoc.

Disease Models & Mechanisms • Supplementary information

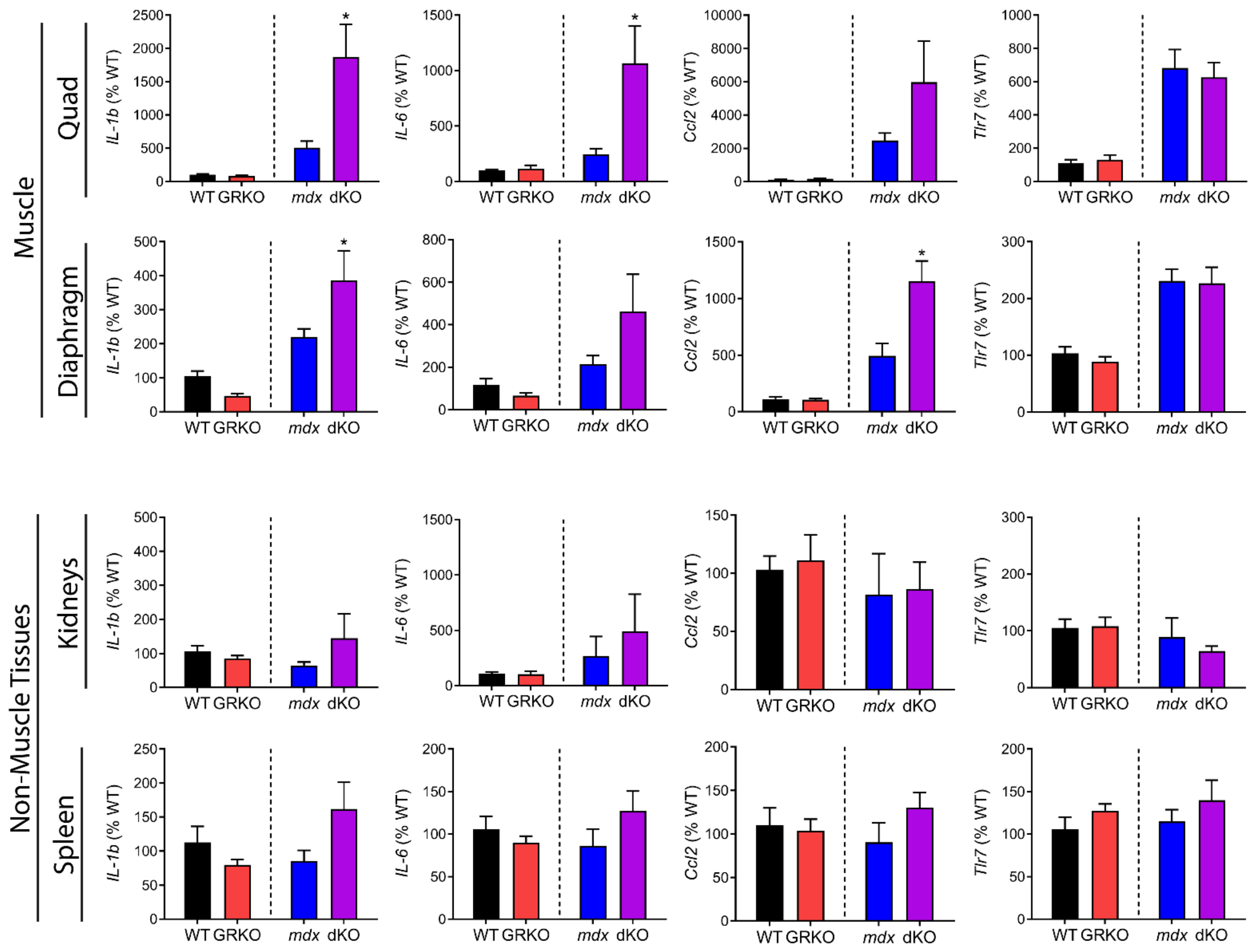

Fig. S5. Extended characterization of inflammatory gene expression. We assayed inflammatory gene expression in muscle (quadriceps and diaphragm) and non-muscle (kidney, spleen) from three-month old mice via qRT-PCR. Gene expression of muscle shows inflammatory gene expression significantly increases for several transcripts in dKO mice versus *mdx* littermate controls. Consistent with chronic inflammation in muscular dystrophy, several of these transcripts are already increased in *mdx* mice versus WT. No significant changes were observed in non-muscle tissues. *n*≥6 per group. *$P \leq 0.05$, **$P \leq 0.005$; unpaired two-tailed *t*-test of Cre-positive vs. control littermates.

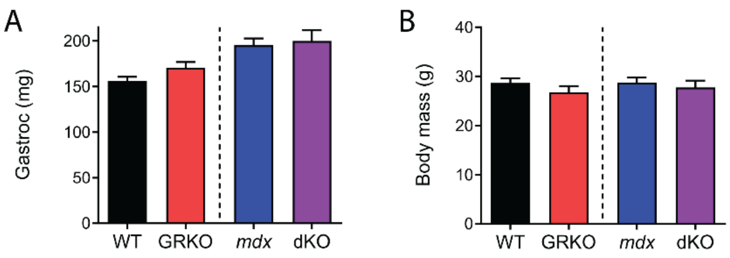

Fig. S6. Gastrocnemius muscle and body mass. At the time of dissection, tissue and mouse weights were obtained. Raw (A) gastrocnemius muscle mass, and (B) body mass values are provided. *n*≥6 per group.

Disease Models & Mechanisms • Supplementary information

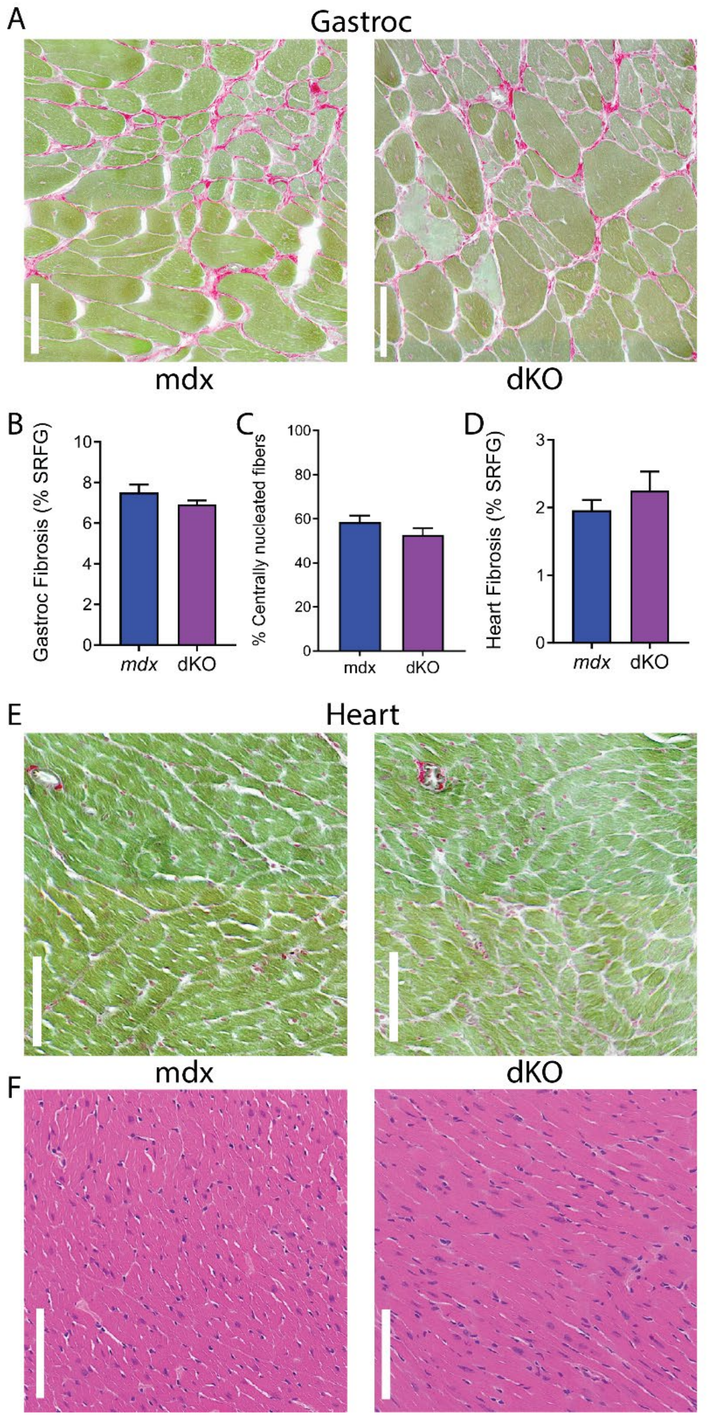

Fig. S7. Histology staining in gastrocnemius and heart muscle. Histological sections of tissues from 3-month old mice were stained with H&E to examine general pathology or Sirius Red Fast Green (SRFG) staining for fibrosis. (A-B) Representative images and quantification of SRFG staining in gastrocnemius muscle. (C) Quantification of centrally nucleated fibers in gastrocnemius muscle; these were scored and calculated from Laminin-stained sections. (D-E) Quantification and representative images of SRFG staining in heart sections. (F) Representative H&E images of heart sections. *n*=3-4 per group. Scale bars: 100 μm.

Disease Models & Mechanisms • Supplementary information

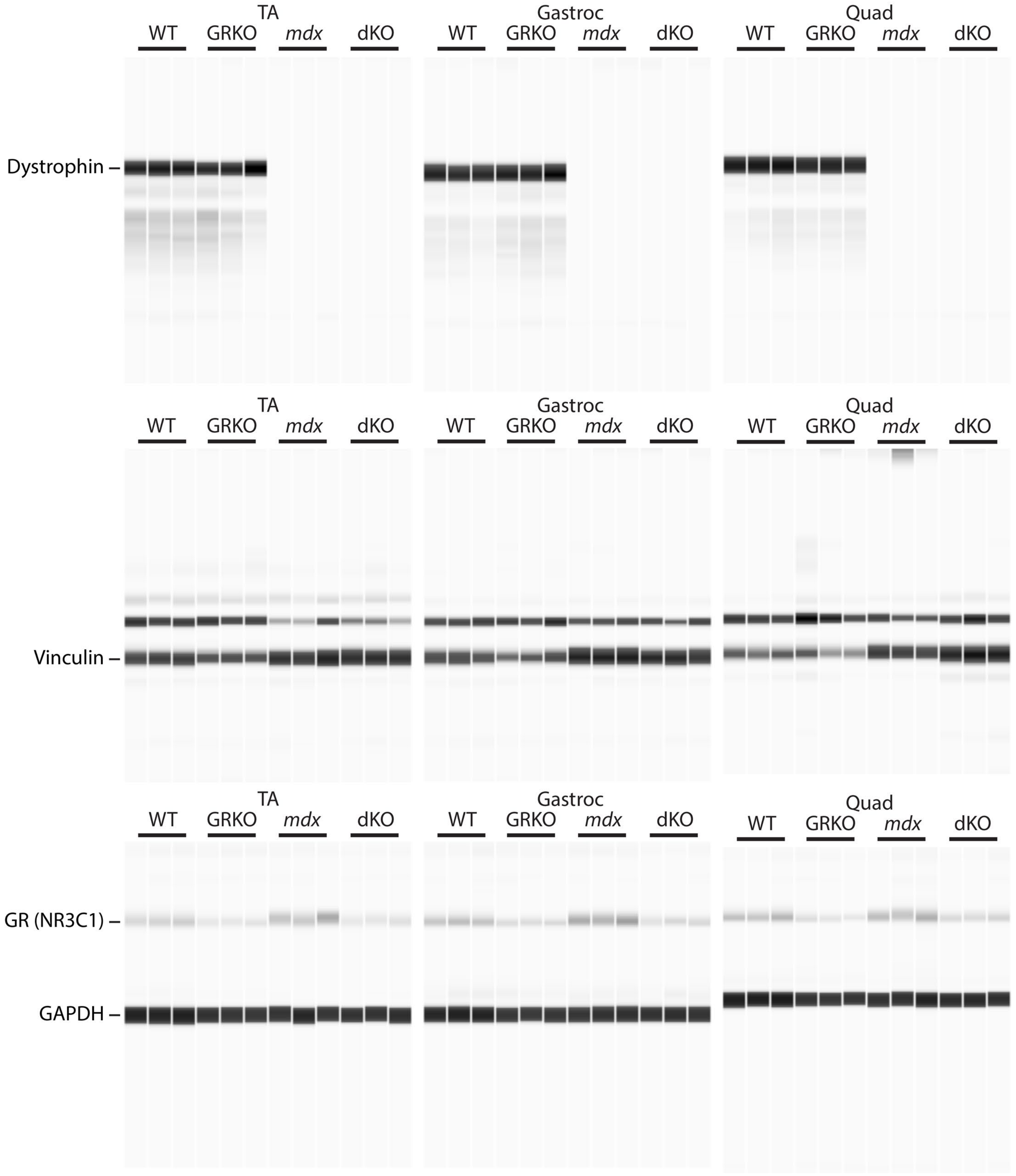

Fig. S8. Full Wes capillary immunoassay images for muscle tissues assayed in Figures 2 and S3. Full Wes capillary immunoassay images provided for muscles found in manuscript Fig. 2A. The Dp71 region of Quad dystrophin was also featured in Fig. S3B. GAPDH, glyceraldehyde-3-phosphate dehydrogenase; Gastroc, gastrocnemius; Quad, quadriceps; TA, tibialis anterior.

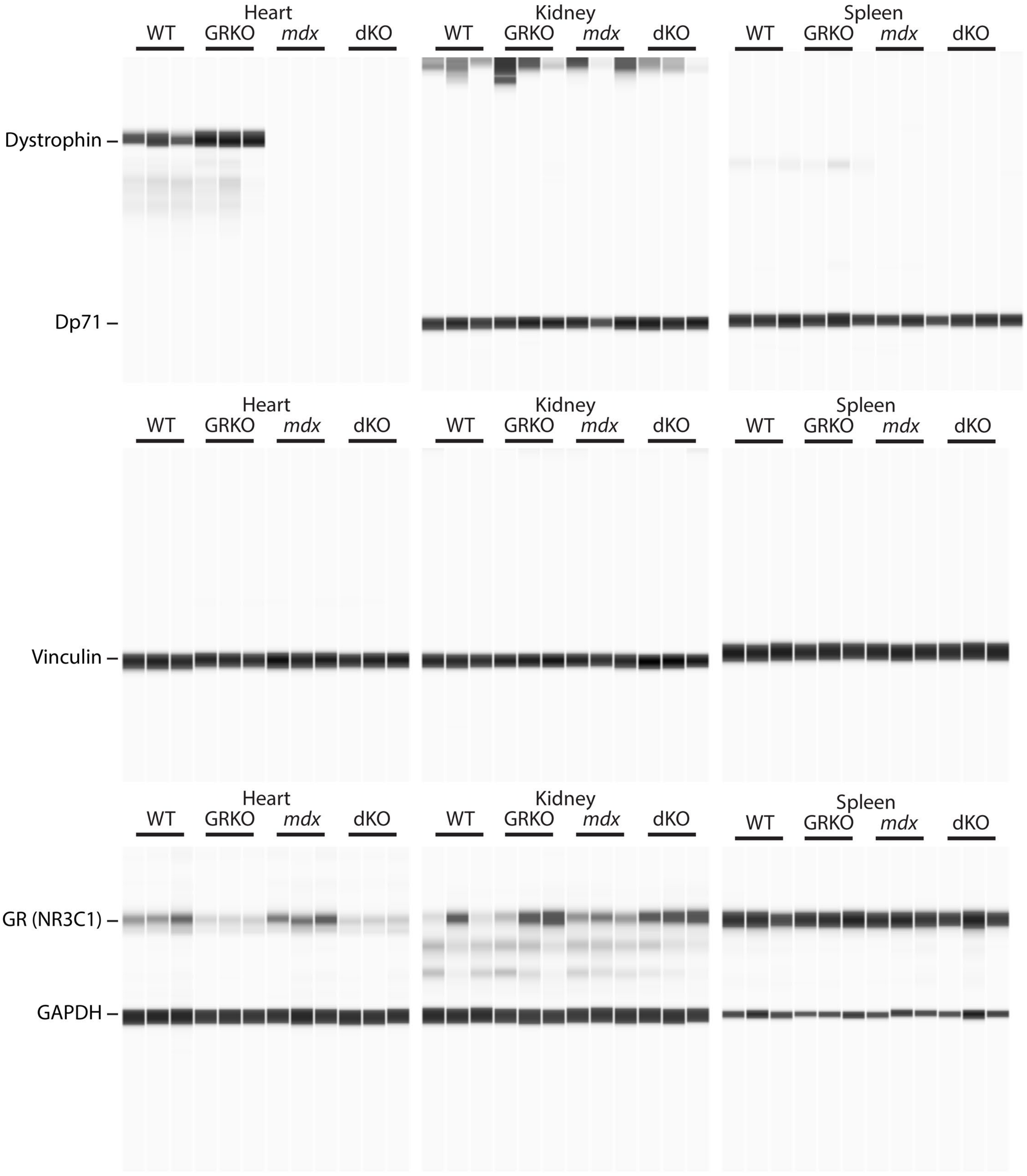

Fig. S9. Full Wes capillary immunoassay images for heart and non-muscle tissues assayed in Figures 2 and S3. Full Wes capillary immunoassay images provided for heart and non-muscle tissues depicted in manuscript Figs 2B and 2C, respectively. The Dp71 region of Kidney dystrophin was also featured in Fig. S3A. GAPDH, glyceraldehyde-3-phosphate dehydrogenase.
